# The synthesis and characterization of giant Calixarenes

**DOI:** 10.1038/s41467-018-07751-4

**Published:** 2019-01-10

**Authors:** Vincent Guérineau, Marion Rollet, Stéphane Viel, Bénédicte Lepoittevin, Ludovic Costa, Pascale Saint-Aguet, Régis Laurent, Philippe Roger, Didier Gigmes, Cyril Martini, Vincent Huc

**Affiliations:** 1 Institut de Chimie des Substances Naturelles, CNRS UPR2301, Université Paris-Sud, Université Paris-Saclay, Avenue de la Terrasse, 91198 Gif-sur-Yvette, France; 2 Aix Marseille Univ, CNRS, ICR, UMR7273, 13397, Marseille, France / Institut Universitaire de France, 75000 Paris, France; 3 LCMT, UMR 6507, ENSICAEN, UNICAEN, CNRS, Normandy University, 14000 Caen, France; 4 Institut de Chimie Moléculaire et des Matériaux d’Orsay (ICMMO) UMR 8182, Univ Paris Sud, CNRS, Université Paris-Saclay, 91405 Orsay, France; 5 Plateforme TECHNOPOLYM, Institut de Chimie de Toulouse, Université Paul Sabatier, 118 Rte de Narbonne, 31062 Toulouse cedex 9, France; 6 Laboratoire de Chimie de Coordination du CNRS, 205 route de Narbonne, 31077 Toulouse Cedex 4, France / Université de Toulouse, UPS, INPT, F-31077 Toulouse Cedex 4, France

## Abstract

Calixarenes are cyclic oligomers obtained by condensation of suitable *p*-functionalised phenols with formaldehyde, usually allowing for the synthesis of the well known small calixarenes (including up to eight phenolic subunits). We report here the discovery of much larger members of this family, exhibiting sizes up to 90 phenolic subunits: the giant calixarenes. These macrocycles are obtained according to simple, easily scalable processes, in yields up to 65%. We show that the formation of these giant macrocycles is favored by an oxygen-containing-group at the para-position of the starting phenol, high concentrations of heavy alkaline bases (rubidium or cesium hydroxides) and long reaction times. A mechanism is proposed to rationalize these observations. These giant macrocycles can also be obtained in the quasi-solid state, opening interesting perspectives in the field of calixarenes chemistry. Along with their intrinsic fundamental interest, these objects are also opening interesting applicative potentialities.

## Abstract (web summary)

Calixarenes are cyclic oligomers obtained by condensation reactions between phenols and formaldehyde and usually include up to eight phenolic subunits. Here the authors demonstrate the synthesis of calixarenes with up to 90 phenolic subunits with up to 65% yield via a scalable process.

## Introduction

Within the macrocyclic compounds family, calixarenes are certainly amongst the most popular members, due to their fascinating chemical and physical properties^1^. Indeed, since its renewal in the late 70’s^1^, calixarene chemistry tremendously expanded and found its way in most fields of molecular chemistry and materials^1–4^. As a consequence, calixarenes are now very popular building blocks in such diverse areas as molecular^5–12^ and supramolecular systems^13–15^ (rotaxanes^16,17^, inclusion complexes^18–20^, solid-state architectures^21^, ionic recognition^1,4,22–24^, synthesis of nanoparticles-based architectures and catalysis^12,25–33^, biological applications^34–37^, and so on.

Despite a sustained interest in the calixarene field, the number of existing calixarenic platforms is still surprisingly small. This is mostly due to the inherent difficulty of calixarene synthesis, especially regarding purification concerns. As a consequence, *p*-(^t^Bu)phenol is to date the only starting product allowing for calixarenes of various sizes to be easily obtained in the pure state on a large scale, thanks to the considerable work of Gutsche and collaborators during the 80’s^1,38–40^. The size of these macrocycles usually ranges from 4 to 8 phenolic repeating units (PRUs), with only very few studies being devoted to larger members (up to 20 PRU^41–46^).

**Fig. 1:**
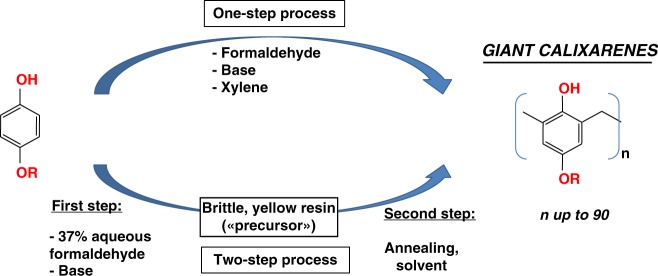
The two base-catalyzed processes used for giant calixarenes synthesis. The conditions used are summarized on the scheme

Calixarenes are generally obtained by mean of a base-catalyzed condensation between a suitably *p*-functionalized phenol with formaldehyde or paraformaldehyde^38,47^. Only a handful of Lewis^48^ or Brönsted^41^ acid-catalyzed syntheses are known. Two different synthetic processes are commonly used. The first one is a one-step process, all the components (phenol/formaldehyde/base) being refluxed in a high boiling solvent (ex. xylene). The second one is a two-steps process, initially optimized by Gutsche and co-workers for the synthesis of *p*-(^t^Bu)calix[4] and [6]arenes^39,40^. In this case, a solid phenol/formaldehyde condensation product is obtained during a first step (the so-called precursor). This solid precursor is subsequently refluxed in a high boiling solvent during a second step, leading to the formation of calixarenes (Fig. 1).

We show here that both the one-step and two-steps processes, used in combination with some specific *p*-functionalized phenols, lead to a surprisingly high yield (up to 65%) of very large cyclic oligomers, the giant calixarenes.

This strongly contrasts with the well-known *p*-(^t^Bu)phenol/formaldehyde/base system, that under the same conditions leads to classical, small sized calixarenes (with 4 to 8 PRU) as the main products^47^. The average size of these giant macrocycles can be easily tuned, notably by a proper choice of the base used as catalyst and its amount. Surprisingly, we found that very large cyclic oligomers can also be obtained according to a quasi-solid-state synthesis, without any kind of stirring/grinding mechanism. This example of a quasi-solid-state synthesis of calixarene suggests a particular reactivity under these conditions. The size range of these molecule, and their large number of functionalization sites make them organic nanoobjects (in the same way as dendrimers). Their interest is reinforced by the fact that both sides of *p*-(benzyloxy)phenol-derived giant calixarenes can be orthogonally functionalized upon hydrogenolysis of the benzyloxy groups^11,27,28^. We thus believe that giant calixarenes are opening a chapter in calixarenes history, and could also be interesting in some areas of nanosciences.

## Results

### Giant Calixarenes discovery

**Fig. 2:**
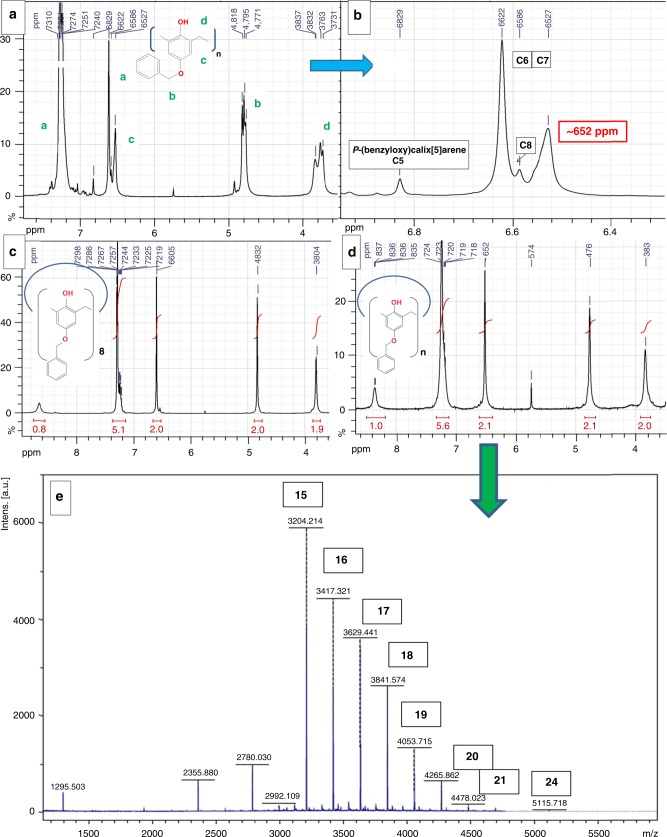
First evidences of giant calixarenes. **a** ^1^H NMR analysis (DMSO-d6) of the crude mixture of calixarenes obtained from a RbOH-catalyzed reaction (0.3 equivalent vs. phenol); **b** Zoom over the hydroquinone protons area; **c** ^1^H NMR spectrum of the first precipitate obtained by hot filtration of the acetone/DMSO solution; **d** ^1^H NMR and **e** MALDI-TOF MS (Na^+^ cationized) of the precipitate obtained upon cooling the acetone/DMSO solution. PRU numbers shown in boxes

This family of compounds was serendipitously discovered during the course of our investigations of the chemistry of *p*-(benzyloxy)calixarenes ^11,27,31,49–52^. During an attempted synthesis of *p*-(benzyloxy)calix[6]arene, we refluxed a mixture of *p*-(benzyloxy)phenol, formaldehyde and RbOH (0.3 equivalent vs. phenol) in xylene. This synthetic process is well known to produce *p*-(^t^Bu)calix[6]arene (74% yield) starting from *p*-(^t^Bu)phenol^38^. To our surprise, the expected *p*-(benzyloxy)calix[6]arene was not obtained in such a high yield. A complex mixture of *p*-(benzyloxy)calixarenes was obtained instead, including *p*-(benzyloxy)calix[5–8]arenes (Fig. 2a). This attribution was made possible by comparing with authentic samples^49,50^, using the most informative hydroquinone-type protons C, Fig. 2a, b.

Interestingly, an additional prominent signal was also observed around 6.52 ppm ((Fig. 2b), highlighted in red, calibration with the signal of DMSO-d6 at 2.49 ppm), whose chemical shifts does not correspond to any *p*-(benzyloxy)calixarene identified so far.

**Fig. 3:**
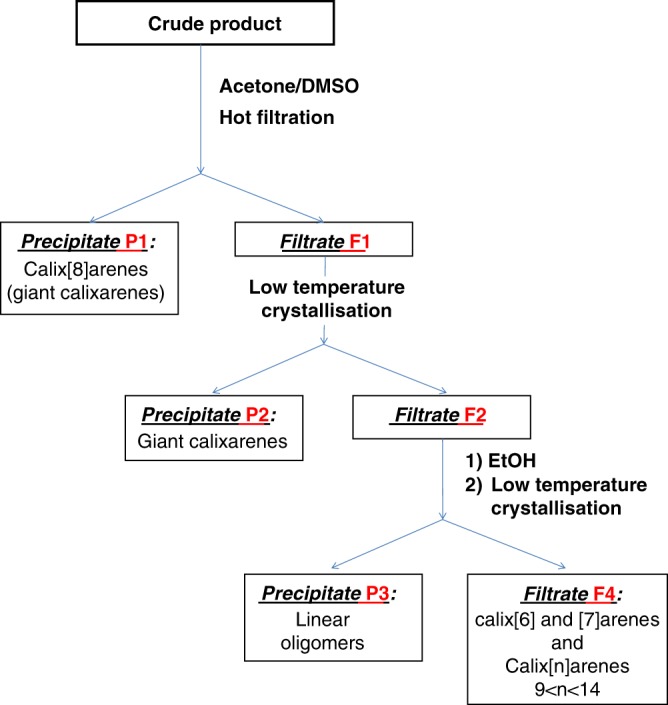
DMSO/acetone-based purification process of giant calixarenes. The different purification fractions are highlighted in red

This unknown calixarene was purified according to purification processes based on a DMSO/acetone or DMSO/EtOH recrystallization. The DMSO-acetone purification process is sketched on Fig. 3 (see also Supplementary Figs 27 and 114). This process first allows for an easy elimination of *p*-(benzyloxy)calix[8]arene by hot filtration (precipitate P1, NMR on Fig. 2c). Second, upon storage of the corresponding acetone/DMSO filtrate F1 at 1 °C, this unknown calixarene was recovered as a microcrystalline precipitate (Fig. 3, precipitate P2) in a 15% yield (NMR on Fig. 2d/e, see also Supplementary Methods). The analysis of the remaining filtrate F2 will be discussed later on.

The very high symmetry of the ^1^H NMR spectrum Fig. 2d is tentatively associated with a calixarene. Moreover, a combined ^1^H/^13^C NMR analysis shows the complete absence of the end groups commonly observed with linear oligomers^53–58^ (see Supplementary Figs 104–108 and 127). Surprisingly, MALDI-TOF MS analysis shows this sample to be indeed exclusively constituted by a mixture of large sized calixarenes, with a broad distribution ranging from 15 to 24 PRU, (M_PRU_ = 212 g/mol) (Fig. 2e and Supplementary Fig. 30). Along with the molecular peaks associated with the different calixarenes, minor signals at M−91 and M+22 atomic mass units (amu) are also observed, associated with photoinduced debenzylation and hydrogen/sodium exchanges, respectively. However, a puzzling feature of the ^1^H NMR spectrum shown on Fig. 2d (and Supplementary Fig. 29) is that despites the fact that this sample is constituted by a mixture of several large calixarenes, only sharp singlets are observed, reminiscent of one single calixarenic size. In order to get a rationale for this phenomenon, a purification process was designed, to obtain pure *p*-(benzyloxy)calix[n]arenes, *n* ranging from 9 to 16 PRUs (Supplementary Note 1). Interestingly, a comparison between the ^1^H NMR spectra of all these different calixarenes shows that chemical shifts are reaching asymptotic values as the ring size increases (Supplementary Fig. 11). This phenomenon has already been observed by Gutsche and co-worker with large *p*-(^t^Bu)calixarenes^41^. Beyond a sufficient size, the local environment around a given phenolic unit remains identical from one calixarene to another. From Supplementary Fig. 11, we can estimate this threshold size around 14 PRU. We can thus consider the presence of these asymptotic chemical shifts (and notably the one around 6.52 ppm for the hydroquinone protons) as characteristic of giant calixarenes. These surprising results prompted us to develop an optimized protocol for giant calixarenes characterization and synthesis. Towards that goal, the influence of the different reaction parameters was systematically investigated

### Giant calixarenes characterizations

Characterizations were performed using a combination of ^1^H and ^13^C NMR (Supplementary Note 2), along with Pulsed Gradient Spin Echo (PGSE) NMR (Supplementary Note 6), Matrix-Assisted Laser Light Desorption-Ionisation Mass Spectrometry (MALDI-TOF MS, (Supplementary Note 3)), Size Exclusion Chromatography (SEC, (Supplementary Note 4)), Dynamic Light Scattering (DLS, (Supplementary Note 8)), and Size Exclusion Chromatography Multiple Angle Light Scattering (SEC-MALS, (Supplementary Note 7)). A pedagogic presentation of each of these different analytical tools is provided in the abovementioned Supplementary Notes. The last three analytical tools are commonly used to determine the average molecular weight of polymers. Their use was made necessary as giant calixarenes are high molecular weight macromolecular objects, obtained as a mixture of ring sizes (as are polymers). Note that the results of both SEC and SEC-MALS experiments will be given as their  peak maximum molecular weight, i.e., from the most intense peak observed on the chromatogram. This corresponds to the most abundant specie in a given sample.

**Fig. 4:**
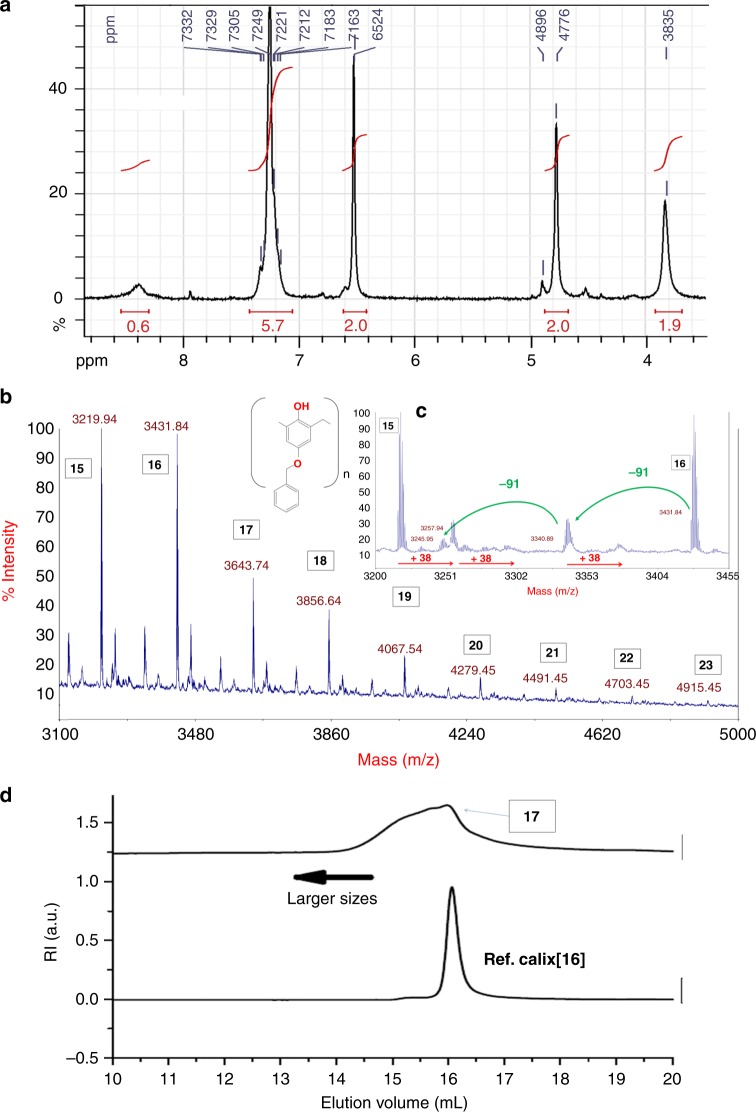
Analysis of a giant calixarenes synthesis (two-steps process, 0.4 equivalent of CsOH vs phenol). **a** ^1^H NMR (DMSO-d6); **b** reflectron-mode MALDI-TOF MS (K^+^ cationized); **c** zoom over the MALDI-TOF MS spectrum (photoinduced debenzylations and hydrogen/metal exchanges shown as green and red arrows, respectively); **d** compared SEC analyses of purified giant calixarenes with a reference sample of pure *p*-(benzyloxy)calix[16]arene. PRU numbers shown in the boxes

**Fig. 5:**
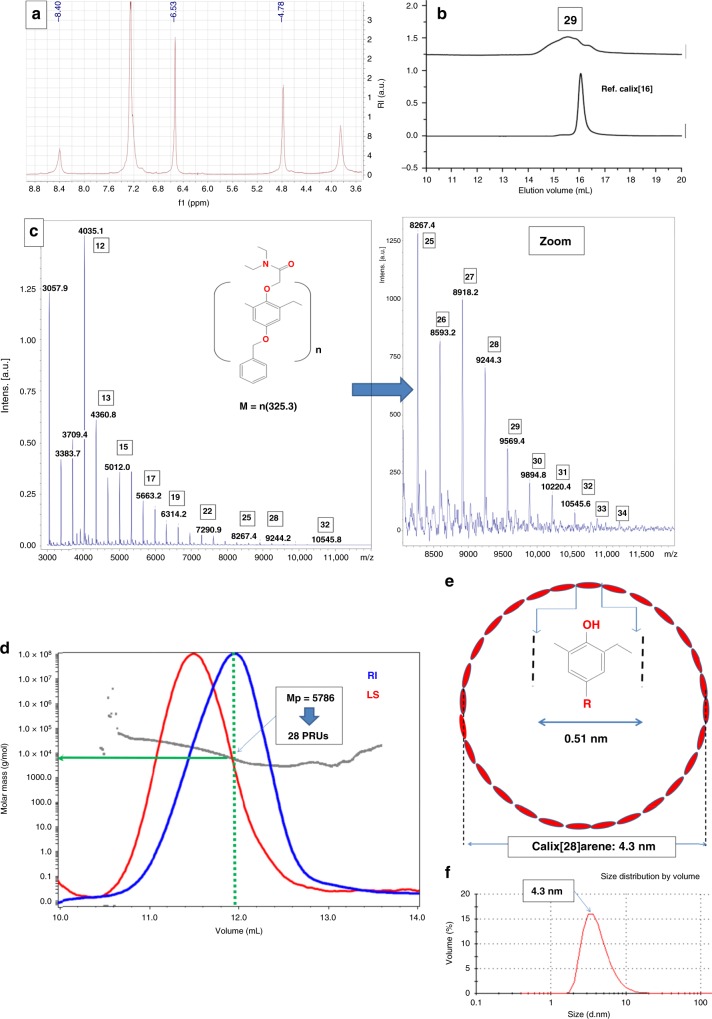
Analysis of giant calixarenes synthesis (one-step process, 0.85 of RbOH vs phenol). **a** ^1^H NMR (DMSO-d6); **b** SEC, and **c** linear mode MALDI-TOF MS analysis of giant calixarenes (Cs^+^ cationized). **d** SEC-MALS analysis ; **e** DLS analysis (size distribution by volume); **f** the geometrical model used to fit DLS results. PRU numbers are shown in the boxes

**Fig. 6:**
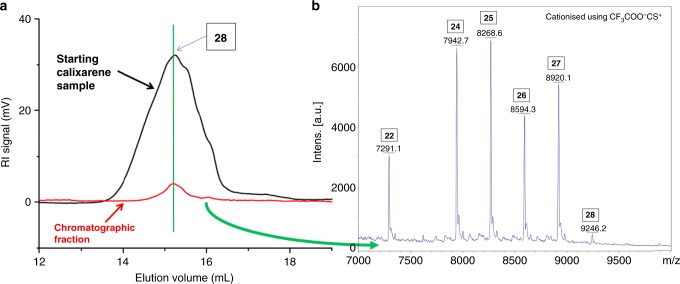
Combined SEC **a** and MALDI-MS **b** analyses of a giant calixarene sample. PRU number shown in the boxes

**Fig. 7:**
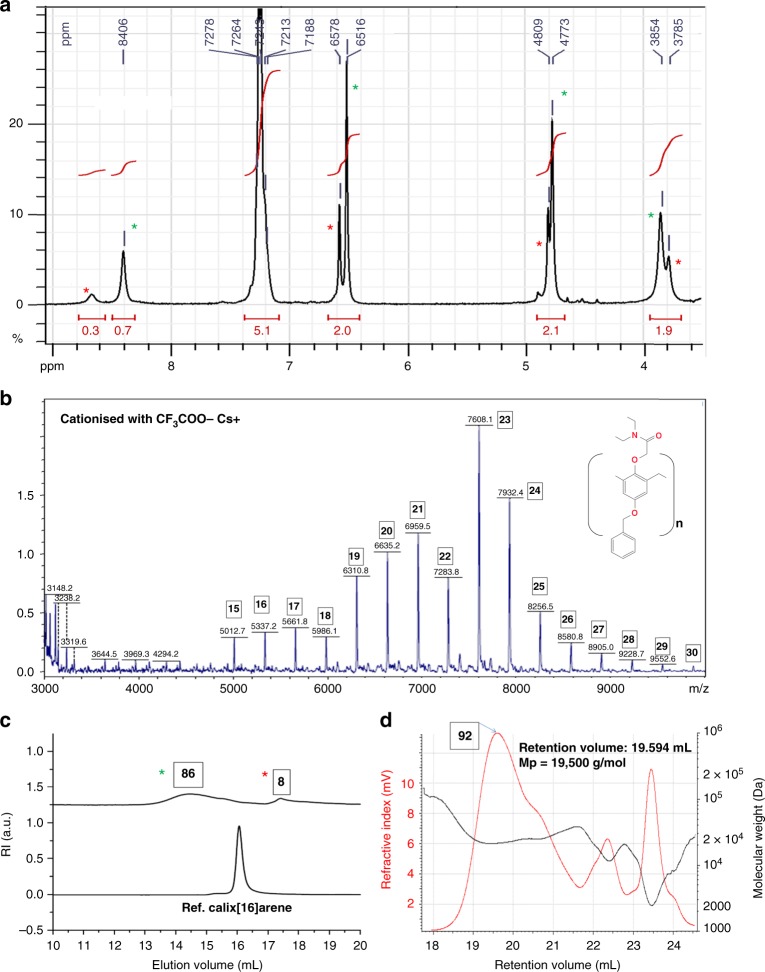
Analysis of a sample with a size range centered around 90 PRUs as the main specie. **a** ^1^H NMR (DMSO-d6); **b** linear mode MALDI-TOF MS (Cs^+^ cationized); **c** SEC; and **d** SEC-MALS analyses of the largest calixarenes isolated (two-step process, 0.8 equivalent of RbOH vs phenol). PRU numbers are shown in boxes. The red and green asterisks are referring to calix[8]arene and giant calixarenes, respectively

Figures 4, 5, 6, 7 are showing the complete analysis of three representative examples of giant calixarenes synthesis, exhibiting different sizes distributions (see Supporting Information for other examples). The first one (Fig. 4, 30% yield, from 100 g of starting phenol) was obtained using the two-steps process with CsOH as a base (0.4 equivalent vs phenol). This sample was purified using the acetone/DMSO purification process (see Supplementary Figs 77–82).

In this case, both SEC and MALDI-TOF MS are giving coherent values, with a SEC-measured on peak size corresponding to a calix[17]arene.

In the same way, Fig. 5 shows the analysis of another purified sample of giant calixarenes, obtained with RbOH as a base (0.85 equivalent vs phenol, see Supplementary Figs 39–48), in a 61% yield (from 122 g of starting phenol) using the one-step process. This sample was purified using the DMSO/EtOH purification process. In this case, SEC analysis shows a much larger size distribution, with an on peak molecular weight centered on a calix[29]arene.

An acetamide-derivatized sample was used for the MALDI-TOF MS analysis in linear mode (Fig. 5c) to increase the signal-to-noise ratio (see Supplementary Note 3 for details).

For these two examples, calixarenes were found to be, by far, the most abundant species, as evidenced by both MALDI-TOF MS and NMR analyses. Indeed, the only significant extra signals observed on the MALDI-TOF MS spectra are belonging to hydrogen/metal exchanges and debenzylations on one hand, or minor amounts of missing acetamide units (M-113) combined with H^+^/M^+^ exchanges on the other hand (Fig. 5c, zoom). As the ring size of the calixarenes increases, linear mode MALDI-TOF MS and SEC analyses are giving different sizes distribution. This is illustrated in the case of the RbOH-catalyzed synthesis (compare Fig. 5b/c). This is not surprising: as detailed in the Supplementary Note 3, the sensitivity of the MALDI-TOF MS analysis rapidly decreases as the molecular weight of the analyzed molecules increases. The amount of the largest species is thus underestimated. However, this analysis shows the absence of significant amounts of linear oligomers. If present, these linear oligomers are indeed easily detected (see Supplementary Figs 104–108 and 127).

In addition, the average molecular weight and the cyclic shape of these molecules were independently confirmed by a combined SEC-MALS (Fig. 5d, see also Supplementary Figs 47 and 48) and DLS (Fig. 5f and Supplementary Fig. 46) study. It is also worth mentioning here that the same scale law was found for both reference samples of calixarenes and giant calixarenes, thus further demonstrating the cyclic shape of the laters (see Supplementary Note 9).

The SEC-MALS chromatogram (Fig. 5d) shows the elution of the products as detected by light scattering (red curve) and refractometry (blue one), after flowing  through a conventional Size Exclusion Chromatography (SEC) column. From the red curve, the instant molecular weight is determined using a mathematical model (gray curve)^59,60^. At the same time, the blue chromatogram shows the elution of the products as detected by refractive index changes. The intercept between the top of this curve (green dashed line), and the gray curve gives the corresponding absolute molecular weight (green arrow). First, a SEC-MALS analysis of reference samples was performed, in order to check the accuracy of the method (see Supplementary Figs 20 and 21).

In the example shown on Fig. 5, this analysis gives an absolute value of the molecular weight corresponding to 28 PRU, in good agreement with the one obtained by SEC (Fig. 5d and Supplementary Figs 47 and 48). Using the simple geometrical model shown on Fig. 5e, this value led to a theoretical diameter of 4.3 nm, that perfectly matches with the experimental one obtained by DLS (4.3 nm, Fig. 5f). Note that the model shown on Fig. 5e does not represent a proposed structure for the calixarene in solution, but only the size of its hydrodynamic volume (see DLS analyses in the Supplementary Note 8 for a more detailed discussion).

Complementary experiments were also performed in order to further confirm the accuracy of our combined SEC/SEC-MALS molecular weight measurements. The purpose here was to obtain a direct matching between SEC/SEC-MALS on one hand, and MALDI MS analyses on the other hand. We first performed an extensive chromatographic work starting from the previously analyzed giant calixarenes synthesis (RbOH, 0.85 equivalent vs. phenol).

This chromatographic work allowed us to obtain a fraction with a SEC-determined molecular weight distribution (Fig. 6a, red chromatogram) centered at the same value (green vertical line) than the on peak one observed for the starting sample (Fig. 6a, black chromatogram, see also Supplementary Fig. 49 for a more detailed description). It means that this chromatographic fraction contains the most abundant species present in the starting calixarenes mixture.

A MALDI-MS analysis of this chromatographic fraction (after (N,N diethyl)acetamide-derivatization) shows that it is composed of a reduced number of giant calixarenes, exhibiting a distribution around 25–26 PRUs (Fig. 6b). This range is close to the one obtained using SEC and SEC-MALS analyses (29 and 28, respectively). This correspondence between SEC/SEC-MALS on one hand and MALDI MS on the other hand confirms the validity of our approach for molecular weights determinations.

Along with the fact that ^1^H and ^13^C NMR spectra show the absence of any end groups (that would be characteristic of linear oligomers), these combined experiments are coherent with a cyclic shape for our compounds. From the hydrodynamic point of view, giant calixarenes can thus be considered (on average) as spheres, the diameter of these spheres corresponding to the one observed for the calixarene in its fully expanded configuration (a more detailed discussion is to be found in the Supplementary Note 8).

Extensive chromatographic workup also allowed for the recovery of a pure sample of a *p*-(benzyloxy)calix[25]arene (see Supplementary  Figs 50–52 for details).

We also compared the SEC-determined molecular weights of giant calixarenes using calixarenes and linear polymers as calibrants. In the later case, we used (poly)methylmetacrylate (PMMA) as a standard, due to its solubility in the solvent used for the analyses (DMF). Interestingly, we found that the PMMA calibration overestimates the molecular weight of giant calixarenes by a factor of about 2. This allows for a rough estimation of molecular weights of giant calixarenes to be easily obtained, providing that a correction factor of about ½ is applied (see Supplementary Note 5 for a detailed discussion).

The largest calixarenic sizes observed so far are to be found in the precipitate obtained from the hot filtration of the acetone/DMSO solution (Fig. 3, precipitate P1). For example, we analyzed the precipitate (22 g, starting from 122 g of phenol, 14% yield) obtained from a RbOH-catalyzed synthesis performed using the two-steps process with 0.8 equivalent of phenol (Fig. 7, see also Supplementary Figs 97–101).

Surprisingly, Fig. 7a shows that the awaited calix[8]arene (red asterisk), is not the main specie. Indeed, most of this sample is constituted by giant calixarenes (Fig. 7a green asterisks), with a size range centered around 90 PRU (Fig. 7c, see also Supplementary Fig. 99).

A SEC-MALS analysis of this sample shows a size range coherent with the previously determined one, centered around 90 PRUs (Fig. 7d, see also Supplementary Figs 99 and 101).

MALDI-TOF MS shows the absence of significant amounts of linear oligomers (Fig. 7b). RbOH seems to be the most efficient base in that regard. For most of the others bases, the main calixarenic specie in this first precipitate is calix[8]arene. The reason for this rubidium effect is not clear yet. Some specific coordination effects may be invoked. Theoretical analyses are required to get a rationale of this phenomenon.

### Size and yield modulation

**Fig. 8:**
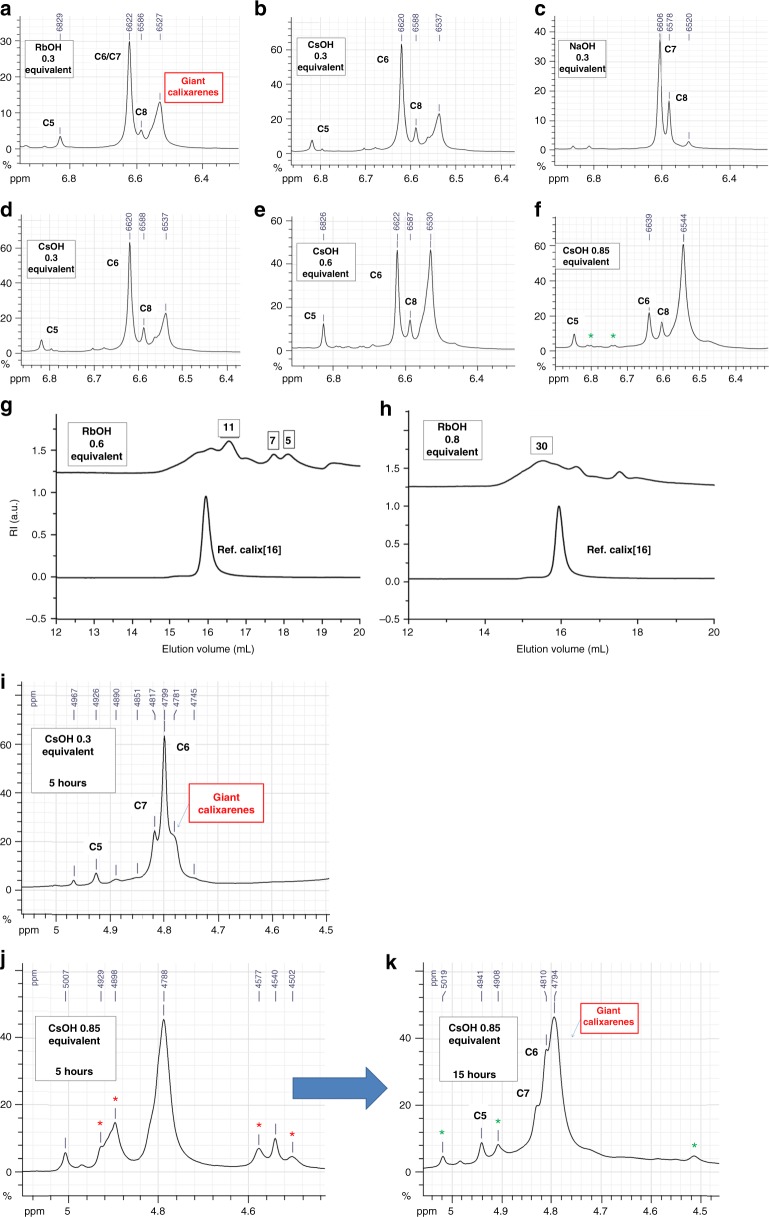
Influence of relevant reaction parameters on the yield and size distribution of giant calixarenes. **a**, **b**, **c** evolution of the ^1^H NMR spectra (DMSO-d6; hydroquinone-type protons area) of crude products obtained with different bases (one-step process used for all examples); **d**, **e**, **f** evolution of the ^1^H NMR chemical spectra of the hydroquinone-type protons of crude products obtained using increasing concentrations of CsOH (one-step process); **g** and **h** SEC comparison of two crude samples obtained using different base/phenol ratio. Number of PRUs shown in the boxes. The green asterisks are highlighting bis(homooxa) *p*-(benzyloxy)calix[4]arene. ^1^H NMR (DMSO-d6) comparison between low (**i**) and high (**j**) base/phenol ratio, and between low (**j**) and high (**k**) refluxing times. Green asterisks: *p*-(benzyloxy)bishomooxacalix[4]arene; red asterisks: linear oligomers

An investigation of the reaction conditions was performed, allowing us to optimize giant calixarenes synthesis. Figure 8 summarizes our most important findings. Figure 8a-c compares the ^1^H NMR spectra of crude reactions products obtained using different bases (0.3 equivalent vs. phenol, one-step process). The lightest bases (for example NaOH) favor the formation of small calix[6–8]arenes. On the opposite, the best yields in giant calixarenes were obtained using the heaviest ones (RbOH and CsOH), regardless of the synthetic process used (one or two-steps). Indeed, the relative intensity of the signal around 6.52 ppm (associated with giant calixarenes) is clearly higher using RbOH and CsOH.

The base/phenol ratio was also shown to exert a strong influence on the yield of giant calixarenes (Fig. 8d-f; see also Supplementary Figs 72–75). Using CsOH, this yield increases from 35 to 70% ongoing from 0.3 to 0.85 equivalent vs phenol (Supplementary Figs 74 and 75), as inferred by the increase of the relative intensity of the peak around 6.52 ppm. The same trends are also observed for the two-steps process (Supplementary Fig. 75), and RbOH as well (Supplementary Figs 72 and 73).

Along with their yield, the size range of giant calixarenes also considerably increases with the base/phenol ratio, regardless of the synthetic process used. For example, ongoing from 0.6 to 0.8 equivalent of RbOH vs phenol, the size shifts from 11 to 30 PRU as shown by the SEC analysis of the crude products (Fig. 8g/h, see also Supplementary Figs 72 and 73).

**Fig. 9:**
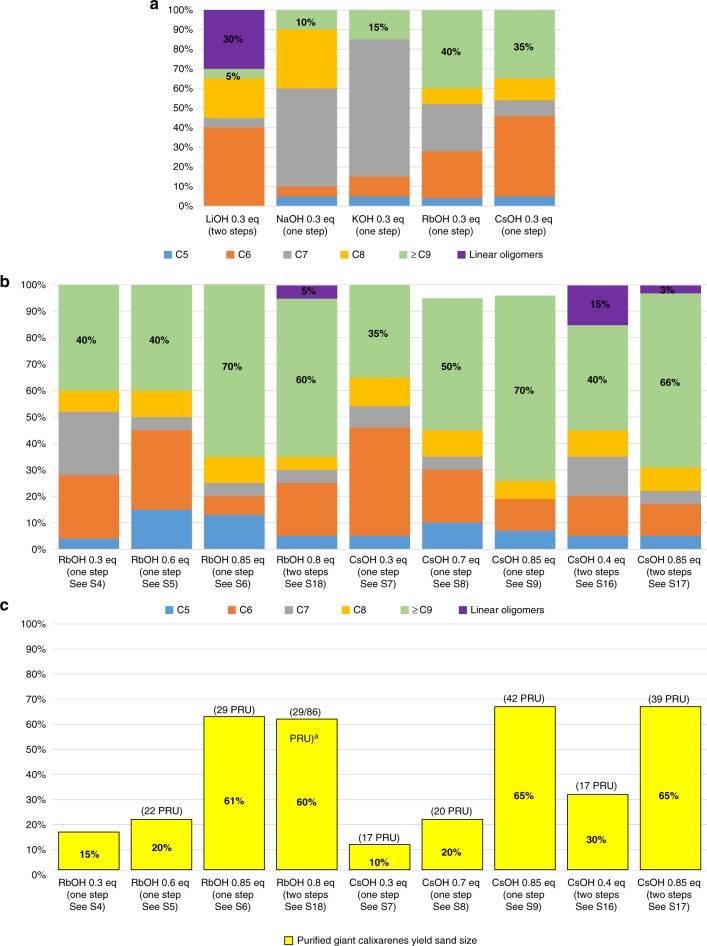
Yields of the different calixarenes as a function of the reaction parameters. **a** effect of the base; **b** effect of CsOH and RbOH/phenol ratio for the one-step and two-steps processes; **c** yield of purified giant calixarenes. Sizes expressed as the number of phenolic repetition units (PRU) determined by SEC from the peak molecular weight. ^a^Combined fractions

We also observed that the reaction time required to fully convert linear oligomers to cyclic ones considerably increases with the base/phenol ratio. Figure 8i-k shows a ^1^H NMR monitoring of calixarenes syntheses using different CsOH/phenol ratios and durations. This monitoring was performed by observing the methylene benzyloxy protons area. Indeed, this area is the most suited for the observation of the end groups associated with linear oligomers. For a 5 hours refluxing time, only calixarenes are observed with a CsOH/phenol ratio of 0.3 (Fig. 8i). On the opposite, over the same period, a considerable amount of linear oligomers are still observed using 0.85 equivalent of base vs. phenol (Fig. 8j, red asterisks, compare with Supplementary Fig. 105). Fifteen hours of reflux in xylene were found to be necessary to fully convert this experiment to calixarenes (Fig. 8k and Supplementary Figs 83–89). Drawn from the analysis of crude products, all these conclusions were fully confirmed by the analysis of the corresponding purified giant calixarenes (Fig. 9c) (see also Supplementary Methods, for examples of giant calixarenes syntheses under different conditions).

Figure 9 summarizes our optimization work (in the case of crude products, yields were determined by ^1^H NMR analyses). Figure 9c shows that both the yield (between 15 and 65%) and size range of giant calixarenes (between 17 to about 90 PRU) can be tuned.

Regarding the two-steps process, we also analyzed in greater details the remaining filtrates obtained during the acetone/DMSO purification of giant calixarenes (Fig. 3, filtrate F2, see also Supplementary Fig. 114). After complete evaporation of acetone (leaving a DMSO-only solution) and addition of ethanol, a microcrystalline precipitate systematically appeared. This precipitate and the complementary filtrate were analyzed separately. As an example, using 0.4 equivalent of CsOH in a two-steps process, a combined ^1^H NMR/MALDI-TOF MS/SEC analysis shows this precipitate to be only constituted of linear oligomers (Supplementary Figs. 105–108). The MALDI-TOF MS analysis (Supplementary Fig. 107) shows the absence of calixarenes. A first family of signals at M_(calix[n]arene)_ + 18 and M_(calix[n]arene)_ + 48 are observed, corresponding to H-(Ar-CH_2_)_n_-CH_2_OH and HOCH_2_-(Ar-CH_2_)_n_-CH_2_OH, respectively (Supplementary Fig. 104a, b). These end groups are also observed on both the ^1^H and ^13^C NMR spectra (Supplementary Figs 105 and 106). Moreover, others families of peaks are also observed, shifted from the previous ones by 30 amu (i.e., insertion of extra –CH_2_-O- units, Supplementary Fig. 104c). These linear oligomers are thus belonging to the well know family of resole-type phenol/formaldehyde polymers^53–58^, commonly observed during the base-catalyzed phenol-formaldehyde condensation.

Moreover, a compared MALDI-TOF MS analysis of calixarenes and linear oligomers of comparable molecular weight shows that both have the same response factor.

An analysis of the filtrate obtained upon filtration of the previously discussed DMSO/ethanol precipitate (Fig. 2, F4) shows it to be constituted exclusively of calix[n]arenes, with *n* ranging from 6 to 14 (two examples shown in Supplementary Figs 109–113).

Thus, the purification process based on acetone/DMSO shows a threshold effect. Importantly, all the calixarenic species with a PRU number < 15 and all the linear oligomers (regardless of their size) are easily separable from giant calixarenes. This in turn constitutes a strong argument to assess the cyclic structure and purity of giant calixarenes.

## Discussion

Our results clearly demonstrate that the *p*-(benzyloxy)phenol/formaldehyde/base system shows a considerable propensity to produce very large cyclic oligomers in high yields. In some cases, their size range expands well above the current record around 20 PRU obtained with *p*-(^t^Bu)calixarenes^41–43,61^. Two questions then arise: why were these giant calixarenes not discovered earlier? Are others *p*-functionalized phenols able to produce large/giant calixarenes in high yields using same conditions? Under high base/phenol ratio (for example 0.5 equivalent of RbOH or CsOH vs. phenol), only small calixarenes were previously described using *p*-(^t^Bu) or others *p*-(alkyl)phenols^47^. However, others products were also mentioned, ascribed to linear oligomers. But the difficulty to discriminate between linear vs. cyclic high molecular weight oligomers leaves the possibility for giant calixarenes for being overlooked. Moreover, we observed that for *p*-(benzyloxy)phenol, reaching the equilibrium composition with high base concentration required unusually long refluxing periods (15 h). This unusually long reaction time is probably due to slowed water removal, in relation with its increased coordination to the metallic cations.

We thus decided to reinvestigate the synthesis of *p*-(alkyl)calixarenes synthesis, using high base concentration and long reaction times. Refluxing for 18 h a xylene solution of *p*-(^t^Bu)phenol/paraformaldehyde/CsOH (1/2/0.8 ratio, one-step process) resulted in a complex mixture of products, where giant calixarenes are indeed observed (Supplementary Fig. 115, one-step process). A giant calixarenes synthesis run with *p*-(heptyl)phenol also resulted in a similar mixture of several products, where very large cyclic oligomers are also observed (Supplementary Fig. 116). However, their amount remains low in both cases, as evidenced by MALDI-TOF MS and ^1^H NMR (Supplementary Figs 115, 116 and 123). A high proportion of linear oligomers is also observed, and the composition in these two cases is far more complex than the one observed using *p*-(benzyloxy)phenol (Supplementary Fig. 123, comparison). On the other hand, ^1^H NMR and MALDI-TOF MS analyses of the crude product obtained from the base-catalyzed condensation of *p*-(octyloxy)phenol with formaldehyde (one-step process, 0.6 equivalent of CsOH vs. phenol) both show that only calixarenes are present (Supplementary Figs 124–126). The formation of giant calixarenes is thus a general phenomenon. Thus, along with high concentrations of heavy alkaline bases (RbOH, CsOH), the presence of an oxygen atom at the *p*-position considerably favors their formation. These puzzling features may be rationalized in three ways. First, large Rb^+^ or Cs^+^ cations are easily dissociated from phenolate anions, thus resulting in net negative charge on the growing intermediate linear oligomers. Electrostatic repulsion may then slow down the last cyclisation steps. This leaves enough time for these oligomers to grow up to large PRU before the final cyclisation. This interpretation is reinforced by the fact that using solvents with a higher polarity than xylene favors the formation of giant calixarenes. As an example, we performed the synthesis of *p*-(benzyloxy)calixarenes in chlorobenzene (Supplementary Figs 117–121), a solvent exhibiting a higher dielectric constant than xylene. The amount of giant calixarenes is increased using this solvent, prone to ease the dissociation of Cs^+^ cations from the corresponding phenolates compared with xylene (Supplementary Fig. 122). Second, the oxygen atoms at the para position is likely to interfere with the cyclisation process, by disturbing the intramolecular hydrogen/coordination bonds networks required to efficiently promote the cyclisation of the intermediate linear oligomers. A longer time will again be left to the intermediate linear oligomers to grow up before cyclisation. Last, the para-oxygen may favor the approach of the phenolic monomers towards the growing linear chain, by coordination to its surrounding cations. This effect is likely to increase the effective concentration of reagents around the growing linear oligomers, thus accelerating their growth rate. All-in-one, the size distribution of giant calixarenes is tunable as a function of both the base used, and its concentration.

Regarding purification concerns, we developed two different purification processes. Both processes are DMSO-based, as this solvent was found to be the only one (with DMF) allowing for true solutions to be obtained (without formation of colloidal suspensions), regardless of calixarene’s sizes. The acetone/DMSO recrystallization process was found to be appropriate if significant amounts of linear oligomers and/or small calixarenes are present in the crude products (as it is usually the case for the two-steps process). The ethanol/DMSO recrystallization process is better suited (due to its simplicity) when lower amounts of linear oligomers and small calixarenes are obtained in the crude products. An interesting feature of the acetone/DMSO recrystallization process is its cutoff effect. As shown above, all the calixarenes with a PRU number below 15 do remain in solution, along with most (if not all) the linear oligomers.

As detailed above, both the one and two-steps synthetic processes are leading to giant calixarenes, and show similar overall trends. However, the one-step process usually gives cleaner results (lower amount of linear oligomers). Regarding the two-steps process, a complete dissolution of the solid intermediate is not always observed. A specific chemistry may thus also occur inside this solid material. This later remark points to the possibility for a specific, solid-state chemistry to occur within the solid precursor during the two-steps synthesis of giant calixarenes. This analysis prompted us to study the possibility to obtain calixarenes using a quasi-solid-state chemistry, i.e., without any dissolution/dispersion of the intermediate solid precursor. Towards that goal, the second annealing step was run using a liquid where the solubility of the solid precursor is negligible at the working temperature (Supplementary Fig. 103). This liquid thus only plays the role of heat transmitter/water remover. Octane or silicone oil proved to be the best ones. This second step was run without any stirring/grinding system (i.e., mechanical stirrer switched off), to avoid any fragmentation/dispersion of the solid, and thus evidence true solid-state reactions. This fundamentally differentiates this approach from the already known ball-milling (grinding) synthesis of calixarenes^62^. After a 20 hours refluxing period at 125 °C in octane, the precursor does not show any dissolution/disintegration or softening features, and thus remains as a brittle solid material all the annealing process long (Supplementary Fig. 103A). For this experiment we used a CsOH/phenol ratio of 0.8. Despites this lack of any visible evolution for this solid, ^1^H NMR monitoring of the composition of this material vs. time revealed deep changes. The spectrum of the yellow precursor at the end of the first step (i.e., before the annealing step) shows its main component to be a phenolic dimer (Supplementary Fig. 103B). At the end of the annealing period in refluxing octane, a complete disappearance of the phenolic dimer is observed, along with the appearance of sharp, symmetric signals characteristic of calixarenes (Supplementary Fig. 103C). Indeed, the MALDI-TOF MS analysis shows this crude material to be constituted nearly exclusively of calixarenes (Supplementary Fig. 103D) including between 9 and 15 PRU. This result shows that a very specific chemistry does indeed occurs within the solid precursor. This is likely to explain the differences observed between the one-step and two-step processes.

We disclose here that some *p*-(alkyloxy)functionalized phenols, used in combination with high concentration of high-Z alkaline bases are prone to produce very large cyclic oligomers in high yields and purity, on a large scale. The systematic study we conducted allows for an easy recovery and a rough tuning of the size of these macrocycles, (up to around 90 PRU), along with high yields. Another characteristic feature is the possibility to obtain these large calixarenic macrocycles according to an original protocol, without any kind of grinding/ball-milling device. Their size makes these giant macrocycles true organic nanoobjects. Thus, along with their ease of functionalization, this family of nanoobjects opens interesting perspectives in nanoscience.

## Methods

All the reagents and solvents were obtained from TCI, and used without any further purification. ^1^ H and ^13^  NMR were recorded on Bruker AC 250, 300 and 360 MHz. All the spectra were recorded in DMSO-d6.

Synthesis: one-step process (see also Supplementary Methods).

### Synthesis

In a typical synthesis, a suspension of 100 g of *p*-(benzyloxy)phenol and 15 g of paraformadehyde in 700 mL of xylene (technical grade) is loaded under argon in a 2-L, three necked flask fitted with a Dean-Stark collector, a magnetic stirrer, and a heating oil bath. The system is then flushed with argon under strong stirring and kept under argon all the synthesis long. The heating bath is then switched on. At 90 °C, the required amount of base (from 0.3 to 0.85 equivalent vs. phenol) is then added as a 50% solution (w/w) in water. The suspension is then refluxed for 6 hours (0.3 equivalent of base) to 15 hours (0.8 equivalent of base).

### Purification process A: DMSO/acetone recrystallization

After cooling the reaction media down to ambient temperature, 500 mL of THF are added, and the suspension is neutralized by slowly adding 1.1 equivalent (vs. initially introduced base) of 37% HCl under strong stirring. The reaction media is then evaporated to dryness. The obtained solid is then suspended in 2 L of methanol under strong stirring for two days and filtered. The cream-colored solid is then suspended in 2 L of acetonitrile under strong stirring for 2 days and filtered. After drying, the precipitate is dissolved in 120 mL of DMSO at 100 °C.  2L of acetone are then rapidly added, and the resulting suspension is hot filtered, leaving a precipitate of p-(benzyloxy)calix[8]arene (combined with giant calixarenes in some cases, especially if RbOH is used). The corresponding filtrate is stored for three days at 1 °C, leaving a microcrystalline precipitate. This precipitate is filtered, washed with a DMSO/acetone solution (10/90 v/v), then pure acetone, and dried under vacuum. Analyses show this precipitate to be only constituted of giant calixarenes, as a mixture of different ring sizes.

### Purification process B: DMSO/EtOH recrystallization

 120 g of neutralized, methanol washed crude product were dissolved in 150 mL of DMSO. A volume of 150 ml of ethanol is then added. This solution is stored overnight at 1 °C. The resulting precipitate is collected by filtration, and dried under vacuum. Analyses show this precipitate to be only constituted of giant calixarenes, as a mixture of different ring sizes.

Note: These syntheses were upscaled up to 400 g of starting p-(benzyloxy)phenol without any difference in the composition/yield. The synthesis and characterisation of p-(benzoyloxy)calixarene is described in the supplementary methods section.

Synthesis: two-steps process (see also Supplementary Methods).

A 2 L three necked round bottomed flask fitted with a mechanical stirrer, a Dean-Stark collector, and a heating mantle is loaded with 104 g of p-(benzyloxy)phenol and 130 mL of 37% formaldehyde. One of the lateral necks is connected via a (closed) glass valve to a water-filled bubbler. The system is then purged with argon. An aqueous 50% weight solution of base (accounting for a base/phenol ration between 0.3 and 0.85) is then added under strong argon flushing, and both the mechanical stirrer and the heating mantle are switched on. The initial thick suspension rapidly turns into a deep yellow clear solution. The reaction is then refluxed for 30 minutes. While keeping the reaction media under reflux, the lateral glass valve is then open while increasing the argon debit, thus enabling a fast argon stream (about 10 bubbles/s) through the flask. This result in a fast removal of water, up to complete evaporation and solidification of the reaction media, resulting in a yellow to orange solid (depending on the initial base loading): the precursor. The mechanical stirrer is then switched off. The duration of this evaporation shows a surprising dependence on base: the higher the amount of base used, the lower the evaporation time (from 30 minutes to 1 h, respectively). A volume of 500 mL of xylene is then added, and the system is then refluxed under strong mechanical stirring for 10 hours under argon. After cooling down to ambient temperature, the resulting thick suspension is neutralized with a solution of 1.2 equivalent of HCl (37%, aq.) vs. the initial amount of base in 700 mL of THF, under strong mechanical stirring for 24 h. The resulting fluid suspension is then evaporated to dryness.

### Purification process A: DMSO/acetone recrystallization

The previously obtained solid is suspended in 2 L of MeOH under strong stirring for 2 days. After filtration and drying, the precipitate is then washed with 2 L of acetonitrile under strong stirring for two days. After filtration and drying, the precipitate is dissolved in 120 mL of DMSO at 120 °C under argon. A volume of 2 L of acetone is then added while hot, and the resulting suspension is hot filtered. The filtrate is then kept at 1 °C for three days, resulting in the formation of a microcrystalline precipitate on the walls of the glassware. This precipitate is recovered by filtration, washed with an acetone/DMSO (90/10) solution, and then with pure acetone and dried under vacuum.

### Purification process B: DMSO/ethanol recrystallization

The crude solid obtained at the end of the second annealing step is suspended in 2 L of MeOH under strong stirring for two days. After filtration and drying, the precipitate is then washed with 2 L of acetonitrile under strong stirring for two days. After filtration and drying, the precipitate is dissolved in 120 mL of DMSO at 120 °C under argon. After cooling down to ambient temperature, 120 mL of ethanol are then added, and the resulting solution is left at 1 °C for three days. The resulting precipitate is then filtered, washed with EtOH, and dried under vacuum.

### Analytical tools

NMR: Otherwise specified all the spectra were recorded in DMSO-d6. See Supplementary Note 1 for details.

MALDI-TOF mass spectrometry: MALDI-TOF MS analyses were performed using an UltrafleXtreme mass spectrometer (Bruker Daltonics, Bremen) or a Voyager DE-sSTR mass spectrometer (AB Sciex, les Ulis France). Acquisitions were performed in reflector or linear positive ion mode. The laser intensity was set just above the ion generation threshold to obtain peaks with the highest possible signal-to-noise (S/N) ratio without significant peak broadening. The mass spectrometer was externally calibrated using PEG. All data were processed using the program FlexAnalysis (Bruker Daltonics, Bremen) or the Data Explorer software package (AB Sciex, les Ulis France).

Trans-2-[3-(4-ter-Butylphenyl)-2-propenylidene] malonitrile (DCTB) was used as the matrix for MALDI-TOF MS. Sodium, potassium, and cesium trifluoroacetate salts were used as cationizing agents. All were of the highest available grade (from Sigma Aldrich Co) and used without further purification. See Supplementary Note 3 for details.

Size Exclusion Chromatography (SEC): The SEC characterization of the giant calixarenes was performed on a PL GPC 120 apparatus equipped with an autosampler and a RI detector, thermostated at 70 °C. 2 PL Resipore columns and a precolumn were used and thermostated at 70 °C. The mobile phase was a mixture of DMF and 0.01 M LiBr delivered at a flowrate of 0.7 mL min^−1^. The samples were prepared with a concentration of 0.25 wt% in a solution containing the mobile phase and 0.25 wt% toluene, used as flow marker. Injection volume was 20 μL.

Polymethylmethacrylate equivalent number-average, weight-average molar masses, and peak maximum molar masses (Mn, Mw, and Mp) and dispersities Ɖ were calculated by means of PMMA calibration curve using PMMA standards from 1.86 to 520.0 Kg.mol^−1^ (Agilent, USA). Calixarènes equivalent number-average, weight-average molar masses, and peak maximum molar masses (Mn, Mw, and Mp) and dispersities Ɖ were calculated by means of calixarenes calibration curve using calixarenes standards from 1.272 to 3.393 Kg.mol^−1^, obtained by the authors after precise purification and characterization (^1^ H NMR, MALDI-MS, SEC-MALS). See Supplementary Note 4 for details.

SEC-MALS molecular weight determination: The SEC-MALS analyses were performed using thermostated columns at 50 °C. Two columns were used: Tosoh Alpha 2500 (exclusion limit: 5 000) + Tosoh Alpha 3000 (exclusion limit: 90 000). The eluent used was a 10 mM LiBr in DMF solution, at a 1 mL/min flowrate. The injected volume was 50 μL.

The detection was made using two detectors:

Differential refractometric detector: Optilab Rex, Wyatt;

Multiangles static light diffusion detector: TREOS Wyatt, 3 angles, laser wavelength = 658 nm.

For the interpretation of the results, the ASTRA VII (Wyatt Technology) software was used.

Alternatively, the SEC-MALS analyses were performed using a MALVERN “VISCOTEC” SEC-MALS 20 apparatus, in THF solutions, using a 35 °C thermostated column. See Supplementary Note 7 for details.

Dynamic Light Scattering (DLS) size determination: The solutions for DLS analyses were prepared by dissolving 15 mg of the calixarene sample in 2 mL of DMSO. The solutions were then stirred for 10 min., filtered (0.2 µ filter), and analyzed. No differences were found in the observed sizes distributions upon repeating the measurement on the same solution 24 h later. The analyses were performed on a Malvern Nano-ZS zetasizer, operating at 633 nm. See Supplementary Note 8 for details.

Pulsed Gradient Spin-Echo NMR exeriments: the experiments were performed at 20 °C on a Bruker AMX 500 500 MHz spectrometer, using DMSO as the solvent. See Supplementary Note 6 for details.

## Supplementary information

Supplementary Information

## Data Availability

The data that support the findings of this study are available from the corresponding author upon request

## References

[1] Gutsche, D *Calixarenes : an introduction*. (The royal Society of Chemistry, Cambridge, 2008).

[2] Vicens J. and Harrowfield J. *Calixarenes in the nanoworld*. (Springer, Dordrecht, Netherlands, 2006).

[3] Asfari Z., Böhmer V., Harrowfield J. and Vicens J. *Calixarenes 2001*. (Kluwer, Dordrecht, 2001).

[4] Mandolini, L. & Ungaro, R. *Calixarenes in action*. (Imperial College Press, London, 2000).

[5] Eggert JPW (2005). Improved synthesis and conformational analysis of an, A,D-1,10-phenanthroline-bridged calix[6]arene. Eur. J. Org. Chem..

[6] Galan H, De Mendoza J, Prados P (2005). Conformational control of calix[6]arenes through multiple bridges.. Eur. J. Org. Chem.

[7] Galan H, Fragoso A, De Mendoza J, Prado P (2008). Synthesis and reactivity of functionalized bridged m-Xylylenedioxycalix[6]arenes. J. Org. Chem..

[8] Otsuka H, Shinkai S (1996). Definitive evidence for inhibition of calix[6]arene ring inversion obtained from a 1,3-xylenyl-bridged chiral calix[6]arene. J. Am. Chem. Soc..

[9] Ross H, Lüning U (1995). Concave reagents based on calixarenes. Angew. Chem. Int. Ed..

[10] Ross H, Lüning U (1997). Concave reagents - 23. Synthesis of a calix[6]arene bridged by a 1,10-phenanthroline. Tetrahedon Lett..

[11] André E (2016). A new, simple and versatile strategy for the synthesis of short segments of zigzag-type carbon nanotubes. Chem. A Eur. J..

[12] Abdellah I. et al. Calix[8]arene as new platform for cobalt-salen complexes immobilization and use in hydrolytic kinetic resolution of epoxides. *Chem. Cat. Chem. ***10**, 4761 (2018).

[13] Arena G. et al. Synthesis of new calixcrowns and their anchoring to silica gel for the selective separation of Cs+ and K+. *Chem. Commun.* 19,2277–2278 (1996).

[14] Mogck O, Böhmer V, Vogt W (1996). Hydrogen bonded homo- and heterodimers of tetra urea derivatives of calix[4]arenes. Tetrahedron.

[15] Mogck O, Pons M, Böhmer V, Vogt W (1997). NMR studies of the reversible dimerization and guest exchange processes of tetra urea calix[4]arenes using a derivative with lower symmetry. J. Am. Chem. Soc..

[16] Casnati A (1995). Synthesis of calix[6]arenes partially functionalized at the upper rim. Tetrahedron.

[17] Arduini A, Ferdani R, Pochini A, Secchi A, Ugozzoli F (2000). Calix[6]arene as a wheel for rotaxane synthesis. Angew. Chem. Int. Ed..

[18] Arduini A (1997). Self-assembled hydrogen-bonded molecular cages of calix[6]arenetricarboxylic acid derivatives. J. Org. Chem..

[19] Coquière D, Cadeau H, Rondelez Y, Giorgi M, Reinaud O (2006). Ipso-chlorosulfonylation of calixarenes: a powerful tool for the selective functionalization of the large rim. J. Org. Chem..

[20] Redon S, Li Y, Reinaud O (2003). Unprecedented selective ipso-nitration of calixarenes monitored by the o-substituents. J. Org. Chem..

[21] McKinlay R, Atwood JR (2007). A hydrogen-bonded hexameric nanotoroidal assembly. Angew. Chem. Int. Ed..

[22] Casnati A (2001). New efficient calixarene amide ionophores for the selective removal of strontium ion from nuclear waste:  synthesis, complexation, and extraction properties. J. Am. Chem. Soc..

[23] Leverd P. C., Dumazet-Bonnamour, I., Lamartine, R., Nierlich, M. Using a large calixarene as a polyalkoxide ligand: tert-butylcalix[12]arene and its complex with the uranyl cation. *Chem. Commun*. **6**, 493–494 (2000).

[24] Thuéry P, Lance M, Nierlich M (1996). Crystal structure of an uranyl/p-tert-butyl calix[6]arene dimer. Supramol. Chem..

[25] Arduini a, Demuru D, Pchini A, Secchi A (2005). Recognition of quaternary ammonium cations by calix[4]arene derivatives supported on gold nanoparticles. Chem. Commun..

[26] Hartlieb KJ, Saunders M, Raston CL (2009). Templating silver nanoparticle growth using phosphonated calixarenes.. Chem. Commun..

[27] Huc V, Pelzer K (2008). A new specifically designed calix[8]arene for the synthesis of functionalized, nanometric and subnanometric Pd, Pt and Ru nanoparticles. J. Colloids. Interface Sci..

[28] Casnati, A., Ferdani, R., Pochini, A. & Ungaro, R. p-(Benzyloxy)calix[8]arene: One-Pot Synthesis and Functionalization. *J. Org. Chem.* **62**, 6236–6239 (1997).

[29] Tshikhudo TR (2005). Molecular recognition by Calix[4]arene‐modified gold nanoparticles in aqueous solution.. Angew. Chem. Int. Ed..

[30] Wei A (2006). Calixarene-encapsulated nanoparticles: self-assembly into functional nanomaterials.. Chem. Commun..

[31] Yousuf Raza (2011). Matrix-dependent cooperativity in spin crossover Fe(pyrazine)Pt(CN)4 nanoparticles. Chem. Commun..

[32] Ray P (2018). IL: Stabilisation of small mono- and bimetallic gold–silver nanoparticles using calix[8]arene derivatives. New. J. Chem..

[33] Huc V (2018). Benzyloxycalix[8]arene: a new valuable support for NHC palladium complexes in C–C Suzuki–Miyaura couplings. Dalton. Trans..

[34] Goodworth K (2011). Synthesis and in vivo biological activity of large-ringed calixarenes against Mycobacterium tuberculosis. Tetrahedron.

[35] Casnati A (1996). Synthesis, antimicrobial activity and binding properties of calix[4]arene based vancomycin mimics. Bioorg. Med. Chem. Lett..

[36] Salvio R (2016). Upper rim bifunctional cone-Calix[4]arenes based on a ligated metal ion and a guanidinium unit as DNAase and RNAase mimics. J. Org. Chem..

[37] Sestito SE (2017). Amphiphilic guanidinocalixarenes inhibit Lipopolysaccharide (LPS)- and Lectin-Stimulated Toll-like Receptor 4 (TLR4) signaling. J. Med. Chem..

[38] Gutsche DC, Dhawan B, No KH, Muthukrishnan R (1981). Calixarenes. 4. The synthesis, characterization, and properties of the calixarenes from p-tert-butylphenol. J. Am. Chem. Soc..

[39] Gutsche CD, Dhawan B, Leonis M, Stewart D (1989). p-tert-Butylcalix[6]arene. Org. Synth..

[40] Gutsche CD, Iqbal M (1989). p-tert-Butylcalix[4]arene. Org. Synth..

[41] Gutsche CD, Stewart DR (1999). Isolation, characterization, and conformational characteristics of p-tert-Butylcalix[9−20]arenes. J. Am. Chem. Soc..

[42] Bavoux C, Baudry R, Dumazet-Bonnamour I, Lamartine R, Perrin M (2001). Large calixarenes: structure and conformation of a calix[16]arene complexed with neutral molecules. J. Incl. Phenom. Macrocycl. Chem..

[43] Perrin M (2001). Crystal structures of two calix[10]arenes complexed with neutral molecules. J. Incl. Phenom. Macrocycl. Chem..

[44] Ferchichi M (2011). The first inexpensive, simplified and large scale synthesis of p-tert-butylcalix[7] and [9]arenes. CheM.

[45] Fleming S (2003). Calixarenes as aryloxides: oligonuclear europium(III) derivatives. Dalton Trans..

[46] Delaigue X (2004). Calixarene complexes of anion-bridged oligouranyl species. Supramol. Chem..

[47] Gutsche CD, Dhawan B, Chen SI (1987). Studies of the formation df calixarenes via condensation of p-alkylphenols and formaldehyde. Makromol. Chem..

[48] Bew SP, Sharma SV (2007). cAn expedient one-pot synthesis of para-tert-butylcalix[8]- and [9]arene. Chem. Commun..

[49] Huc V, Guerineau V (2010). C3v (Trimethyl) p-(Benzyloxy)calix[6]arene: a versatile platform for the synthesis of functionalized C3v Calix[6]arenes. Eur. J Organic Chem..

[50] Huc V (2010). p‐(Benzyloxy)calix[8]arene synthesis revisited: p-(Benzyloxy)calix[4], p-(Benzyloxy)calix[5], p-(Benzyloxy)calix[7], and p-(Benzyloxy)bis(homooxa)calix[4]arenes. Eur. J. Org. Chem..

[51] Leverd P, Huc V, Palacin S, Nierlich M (2000). Octa(p-hydroxy)octakis(propyloxy)calix[8]arene: the first crystal structure of a p-Hydroxy alixarene. J. Incl. Phenom. Macrocycl. Chem..

[52] Levered PC, Huc V, Palacin S, Nierlich M (2000). Crystal structure of octa(p-hydroxy)octakis(propyloxy)calix[8]arene—acetone (1/4), C80H96O16 · 4CH3COCH3. Zeitschrift fur Kristallographie.

[53] Masci B, Thuery P (2005). Synthesis of homooxacalixarenes with 5 and 10 phenol units and crystal structure of their complexes with uranyl ions. New. J. Chem..

[54] Pizzi A, Pasch H, Simon C, Rode K (2004). Structure of resorcinol, phenol, and furan resins by MALDI‐TOF mass spectrometry and 13C NMR. J. Appl. Polym. Sci..

[55] Lin-Gibson S (2002). Cresol novolak/epoxy networks: synthesis, properties and processability. Polymer.

[56] Mandaland H, Ha AS (1997). M.A.L.D.I.-T.O.F. mass spectrometry characterization of 4-alkyl substituted phenol-formaldehyde novalac type resins. Polymer..

[57] Hanton SD (2001). Mass spectrometry of polymers and polymer surfaces. Chem. Rev..

[58] Yamagishi TA, Nomoto M, Yamashita S, Yamazaki T, Nakamoto Y (1998). and Ishida S-i: characterization of high molecular weight novolak. Macromol. Chem. Phys..

[59] Zimm B (1948). The scattering of light and the radial distribution function of high polymer solutions. J. Chem. Phys..

[60] Zimm B (1948). Apparatus and methods for measurement and interpretation of the angular variation of light scattering; preliminary results on polystyrene solutions. J. Chem. Phys..

[61] Stewart DR, Gutsche CD (1993). The one-step synthesis of *p*-tert-butylcalix[5]arene. Org. Prep. Proced. Int..

[62] Atwood JL, Hardie MJ, Raston CL, Sandoval CA (1999). Convergent synthesis of p-Benzylcalix[7]arene: condensation and UHIG of p-Benzylcalix[6 or 8]arenes. Org. Lett..

