# Supplementary material for: The synthesis and characterization of giant Calixarenes
Source: Nat Commun. 2019 Jan 10;10:113. doi: 10.1038/s41467-018-07751-4 (PMC6328600; doi:10.1038/s41467-018-07751-4)
Supplement: Supplementary file 1 — Supplementary Information [file 41467_2018_7751_MOESM1_ESM.docx]

**The Synthesis and Characterisation of Giant Calixarenes**

Vincent Guérineau, et al.

**Supplementary Information**

**Table of contents**

**Supplementary Note 1:** Purification process and characterisations of reference calix[n]arenes (9<n<16)……….......................................................................................... .***9***

Supplementary Figure 1: …………………………………..………………….9

Supplementary Figure 2: ……………………………………..…………...…10

Supplementary Figure 3: ………………………………………………….…11

Supplementary Figure 4: ……………………………………………..……..12

Supplementary Figure 5: ………………………………………..…………..13

Supplementary Figure 6: …………………………………………..………..14

Supplementary Figure 7: …………………………………………………….15

Supplementary Figure 8: …………………………………………………….16

Supplementary Figure 9: …………………………………..………………..17

Supplementary Figure 10: …………………………………..………………18

**Supplementary Note 2:** ^1^H/^13^C NMR…………………………………………….……………19

Supplementary Figure 11: …………………………..………….……………19

**Supplementary Note 3:** MALDI-TOF MS analysis…………..……....……………….…….…20

**Supplementary Note 4:** Size Exclusion Chromatography (SEC) analysis………………….…21

Supplementary Figure 12: ………………………………………………...…21

Supplementary Figure 13: ……………………………………………...……22

**Supplementary Note 5:** Compared SEC analysis using calixarenes or PMMA calibrants:…23

Supplementary Figure 14: ……………………………………………..……23

Supplementary Table 1: ………………………………………………..…....24

Supplementary Figure 15: ……………………..……………………………25

Supplementary Figure 16: ……………………………..……………………26

**Supplementary Note 6:** PGSE NMR analysis………………….…………………………….…28

Supplementary Table 2: ………..……………………………………………28

Supplementary Figure 17: ………...…………………………………………28

**Supplementary Note 7:** Size Exclusion Chromatography - Multiple Angle Light Scattering analysis (SEC-MALS)…….…………………………………………………………..…………….30

Supplementary Figure 18: ……………………………………………..…….30

Supplementary Figure 19: …………………………………………………...31

Supplementary Figure 20: …………………………………………………..32

Supplementary Figure 21: …………………………………………………..33

Supplementary Figure 22: …………………………………………………..34

**Supplementary Note 8:** Dynamic Light Scattering analysis………………………..…………..36

Supplementary Figure 23: …………………………...………………………36

Supplementary Figure 24: …………………………………………………...38

**Supplementary Note 9:** Study of the evolution of the hydrodynamic radius of giant calixarenes as a function of their molecular weight: Scale Law determination………………38

Supplementary Figure 25: …………………………………...………………38

Supplementary Figure 26: …………………………………………………...39

**Supplementary methods: ……….……………..…………….………....40**

**General:**…………………………………………………………………………..………...40

**Giant calixarenes: one-step synthetic process……………....…………40**

Synthesis and purification……….………………………..……………………..………………….40

Supplementary Figure 27: ………………………...…………………………41

**One-step process: RbOH-catalysed reaction:…………………………..……………......42**

RbOH 0.3 equivalent vs. phenol…………….………………..……………..…………………..42

Supplementary Figure 28: ……………………………………...……………42

Supplementary Figure 29: ………………………………………….……..…43

Supplementary Figure 30: ………………………………………………...…44

RbOH 0.6 equivalent vs. phenol: ……..……………………….…..……………..………..45

Supplementary Figure 31: …………………………………………………..45

Supplementary Figure 32: ………………………………………………...…46

Supplementary Figure 33: ………………………………………………...…47

Supplementary Figure 34: ………………………………………………..…48

Supplementary Figure 35: …………………………………………………..49

Supplementary Figure 36: ………………………………………………...…50

Supplementary Figure 37: ………………………………………………...…51

Supplementary Figure 38: …………………………………………………...52

RbOH 0.85 equivalent vs. phenol……..……………………………………..………….…….……53

Supplementary Figure 39: …………………………………………………...53

Supplementary Figure 40: ……………………..……………………………54

Supplementary Figure 41: ………………………………………………..…55

Supplementary Figure 42: ………………………………………………..…56

Supplementary Figure 43: …………………………………………………..56

Supplementary Figure 44: …………………………………………………..57

Supplementary Figure 45: ………………………………………………..…58

Supplementary Figure 46: ………………………………………………..…59

Supplementary Figure 47: ………………………………………………...…60

Supplementary Figure 48: ………………………………………………...…61

combined SEC and MALDI-MS analyses of giant calixarenes…………….………………………62

Supplementary Figure 49: …………………………………………………...62

*p*-(benzyloxy)calix[25]arene……………………………………………………………………......64

Supplementary Figure 50: ……………………………………………………64

Supplementary Figure 51: …………………………………………………...64

Supplementary Figure 52: …………………………………..………………65

**One-step process: CsOH-catalysed reaction:………….…………...…………………...66**

CsOH 0.3 equivalent vs. phenol………………..……………………….………...………………...66

Supplementary Figure 53: ……………………………...……………………66

Supplementary Figure 54: ………………………………..………………….67

Supplementary Figure 55: …………………………………………………...67

Supplementary Figure 56: ………………………………………………..…68

CsOH 0.7 equivalent vs. phenol………………………..……………….……...…………………..69

Supplementary Figure 57: ………………………………...…………………69

Supplementary Figure 58: …………………………...………………………70

Supplementary Figure 59: ……………………...……………………………70

Supplementary Figure 60: ………………………..…………………………71

Supplementary Figure 61: …………………………..………………………72

Supplementary Figure 62: ………………………..…………………………72

CsOH 0.85 equivalent vs. phenol……………………..……………...…...……………….………73

Supplementary Figure 63: …………………………..………………….……73

Supplementary Figure 64: ……………………………………………...……74

Supplementary Figure 65: ………………………………………………..…75

Supplementary Figure 66: ………………………………………………..…76

Supplementary Figure 67: ………………………………………………...…77

Supplementary Figure 68: …………………………………………………..77

Supplementary Figure 69: ………………………………………………..…78

Supplementary Figure 70: ………………………………………………..…79

Supplementary Figure 71: ………………………………………………..…80

Supplementary Figure 72: ………………………………………………..…81

Supplementary Figure 73: ………………………………………………..…82

Supplementary Figure 74: ………………………………………………...…83

**Giant calixarenes: two-step process: ………..………...………………84**

Synthesis and purification………………….…………………………….…………………………84

Supplementary Figure 75: ………………………………………..…………86

Supplementary Figure 76: ………………………………………………..…87

**Two-steps process: CsOH catalysed reactions: ….………………………………..…….88**

CsOH 0.4 equivalent vs. phenol………...….………………………………………...……………..88

Supplementary Figure 77: ………………………………………………...…88

Supplementary Figure 78: ………………………………………………...…89

Supplementary Figure 79: ………………………………………………...…89

Supplementary Figure 80: ………………………………………………..…90

Supplementary Figure 81: ………………………………………………..…91

Supplementary Figure 82: …………………………………………………..92

CsOH/phenol = 0.85…….…………………………………………...………….…………………..93

Supplementary Figure 83: ………………………………………………...…93

Supplementary Figure 84: ………………………………………………..…94

Supplementary Figure 85: ………………………………………………..…94

Supplementary Figure 86: ………………………………………………..…95

Supplementary Figure 87: ………………………………………………..…96

Supplementary Figure 88: ………………………………………………..…97

Supplementary Figure 89: ………………………………………………..…97

**Two-steps process: RbOH catalysed reactions………....…...………...………………....98**

RbOH/phenol = 0.8…………………………………………………………………………………98

Supplementary Figure 90: ………………………………...…………………98

Supplementary Figure 91: …………………………………………………...99

Supplementary Figure 92: …………………………………………………...99

Supplementary Figure 93: ………………………………………………….100

Supplementary Figure 94: ………………………………………………….100

Supplementary Figure 95: ………………………………………………….101

Supplementary Figure 96: ………………………………………………….102

**DMSO/acetone crystallization - hot filtration precipitate analysis.**…………………………………….………………………………………..……103

Supplementary Figure 97: ………………………………………………….103

Supplementary Figure 98: ………………………………………………….104

Supplementary Figure 99: ………………………………………………….105

Supplementary Figure 100: ………………………………………..………106

Supplementary Figure 101: ………………………………………..………107

Supplementary Figure 102: ………………………………………………..108

**Solid-state synthesis………...………………………………………….109**

Supplementary Figure 103: ……………………………………………...…110

**Linear oligomers recovery……..…………………………...………....111**

Supplementary Figure 104: ……………………………………………..…111

Supplementary Figure 105: ………………………………………………..112

Supplementary Figure 106: ……………………………………………..…112

Supplementary Figure 107: ……………………………………………...…113

Supplementary Figure 108: ……………………………………………..…114

**Calixarenes recovery…….…………………………………….………115**

**Example 1: calixarenes recovery from a two-steps process........….………………......115**

Supplementary Figure 109: ……………………………………………...…115

Supplementary Figure 110: ……………………………………………...…116

Supplementary Figure 111: ………………………….…………………..…117

**Example 2: calixarenes recovery from a solid-state synthesis………….…………..…118**

Supplementary Figure 112: ………………………………..………………118

Supplementary Figure 113: ………………………………………...………119

Supplementary Figure 114: ………………………………………..………120

**Giant calixarenes synthesis from *p*-(tBu)phenol (one-step process)..121**

Supplementary Figure 115: ……………………………………...…………121

**Giant calixarenes synthesis from *p*-(n-heptyl)phenol (two-steps process)…………………………………………………………………122**

Supplementary Figure 116: ……………………………………...…………123

**Synthesis using chlorobenzene as a solvent ….………………………124**

Supplementary Figure 117: ……………………………………………..…124

Supplementary Figure 118: ……………………………………………..…125

Supplementary Figure 119: ……………………………………………..…126

Supplementary Figure 120: ……………………………………………..…127

Supplementary Figure 121: ……………………………………………..…128

Supplementary Figure 122: ………………………………………………..129

Supplementary Figure 123: ……………………………………………..…130

***p*-(octyloxy)phenol…...………………………………………………...131**

Supplementary Figure 124: ……………………………………………......131

Supplementary Figure 125: ……………………………………………..…132

Supplementary Figure 126: ……………………………………………..…132

**MALDI-MS analysis of a Giant Calixarenes Synthesis**

Supplementary Figure 127: ……………….………………………………….…133

**Supplementary References:….………………………………………..134**

**Supplementary Note 1: purification process of calix[n]arenes (9<n<16)**

Starting from 6.2g of the crude product obtained from the RbOH 0.3 equivalent-catalysed reaction, the following figure details the purification process leading to the obtention of reference samples of pure p-(benzyloxy)calix[n]arenes. The purification process involves the successive washings and chromatographic steps shown on the following figure. This led to the obtention of pure samples of *p*-(benzyloxy)calixarenes, shown in the red boxes. These pure samples were used as references for the molecular weight determinations.

**Supplementary Figure 1: overview of the purification process of calix[9🡪16]arenes.** The pure, reference calixarene samples obtained are shown in the red boxes.


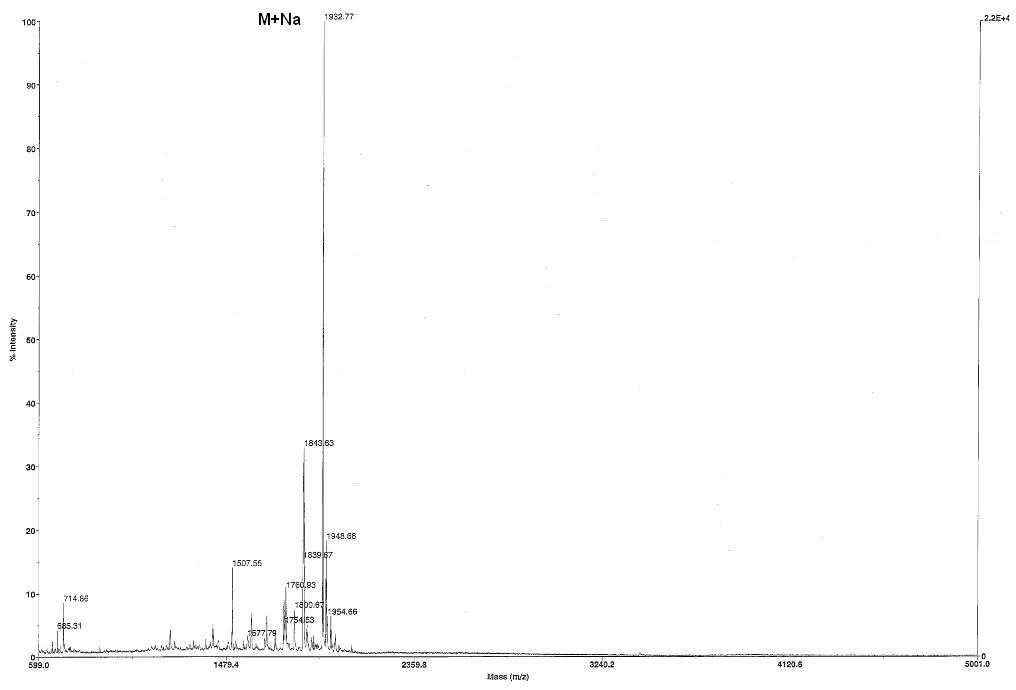


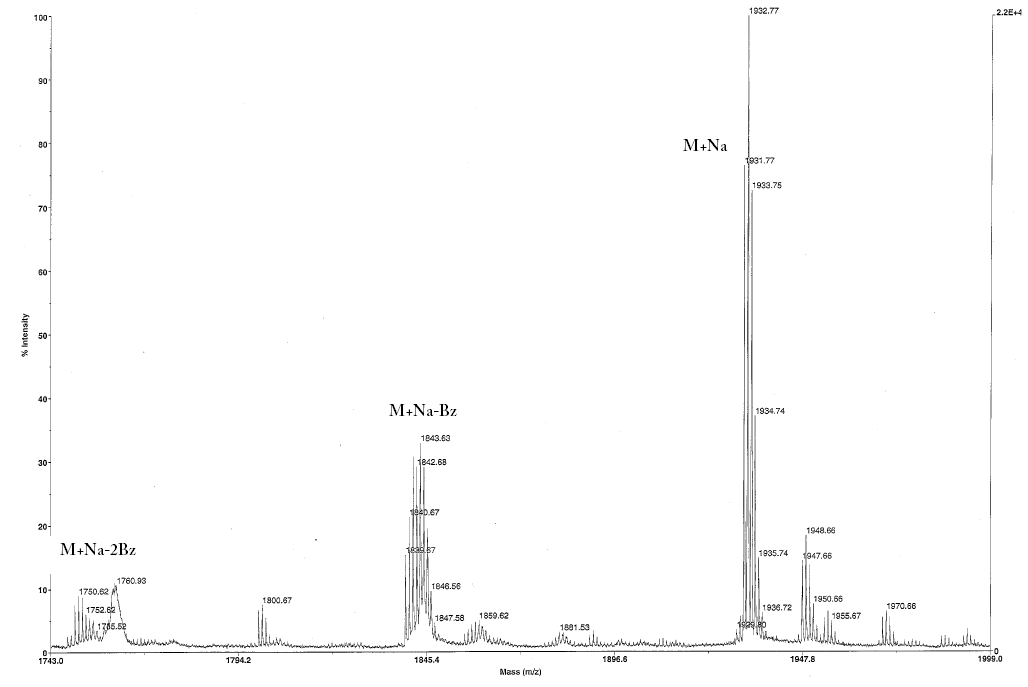


**M+Na-2Bn**

**M+Na-Bn**

**Supplementary Figure 2: MALDI-MS analysis of fraction F6b (*p*-(benzyloxy)calix[9]arene)**


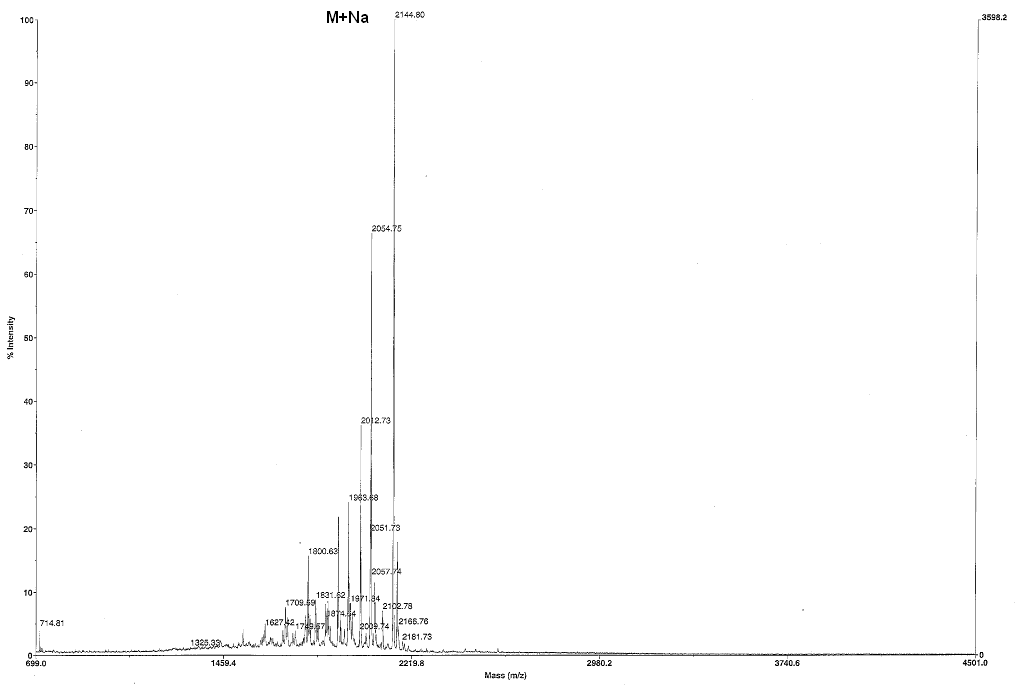

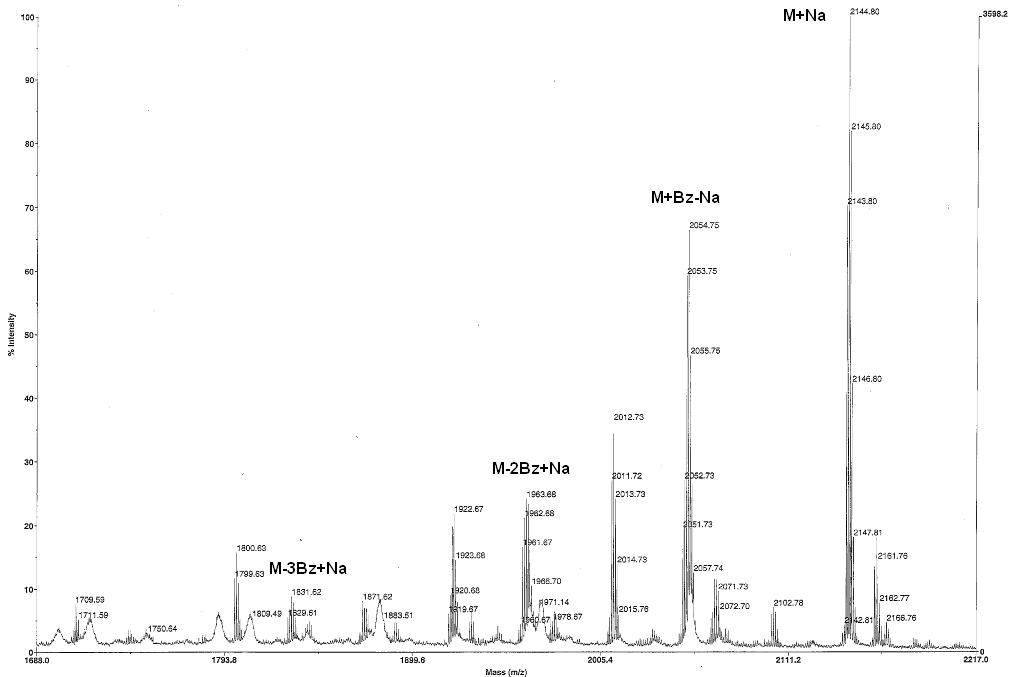


**M+Na-3Bn**

**M+Na-2Bn**

**M+Na-Bn**

**Supplementary Figure 3: MALDI-MS analysis of fraction P7 (*p*-(benzyloxy)calix[10]arene)**


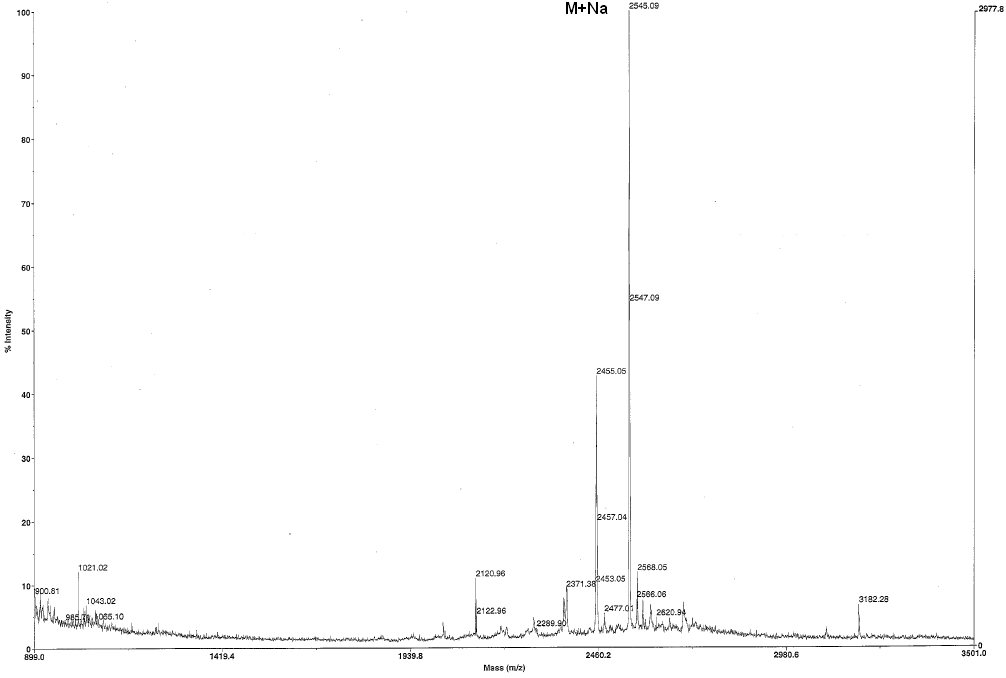

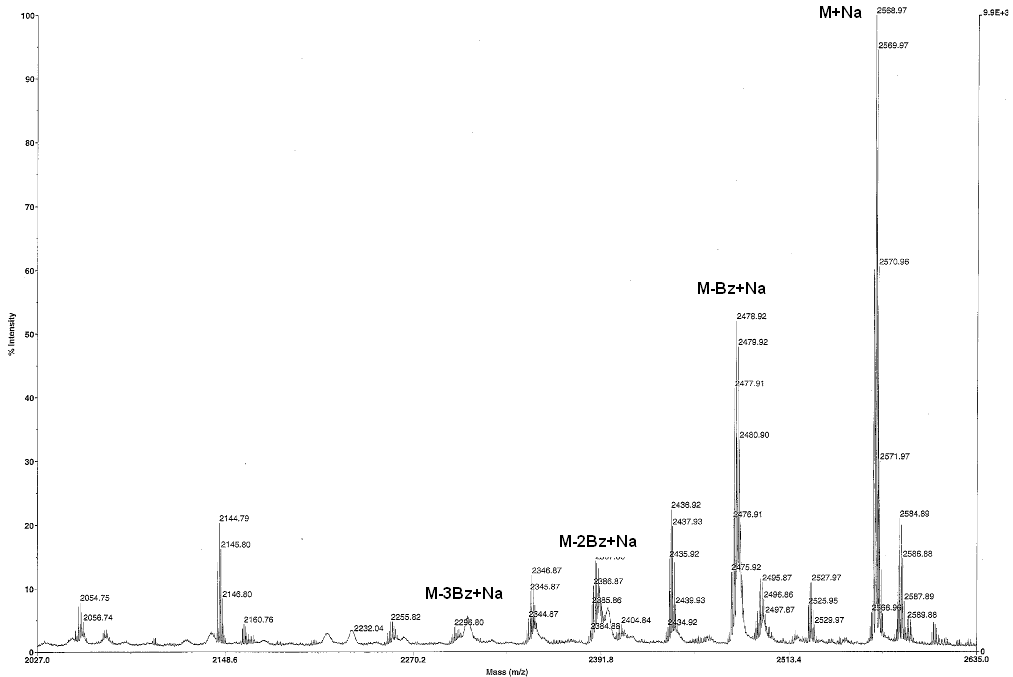


**M+Na-3Bn**

**M+Na-Bn**

**M+Na-2Bn**

**Supplementary Figure 4: MALDI-MS analysis of fraction P10 (*p*-(benzyloxy)calix[12]arene)**


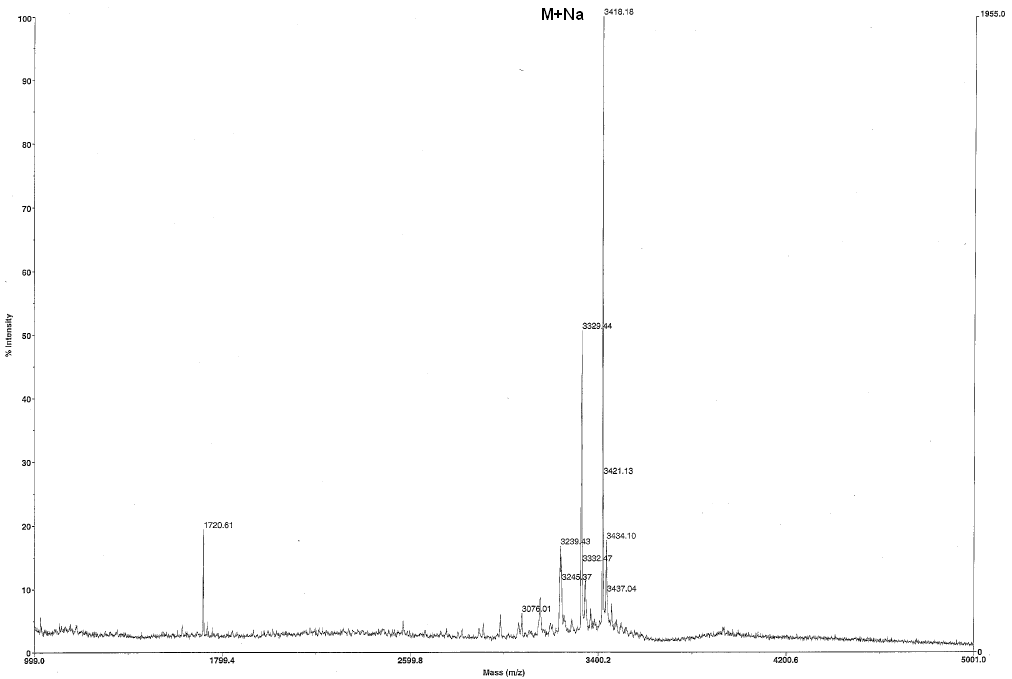

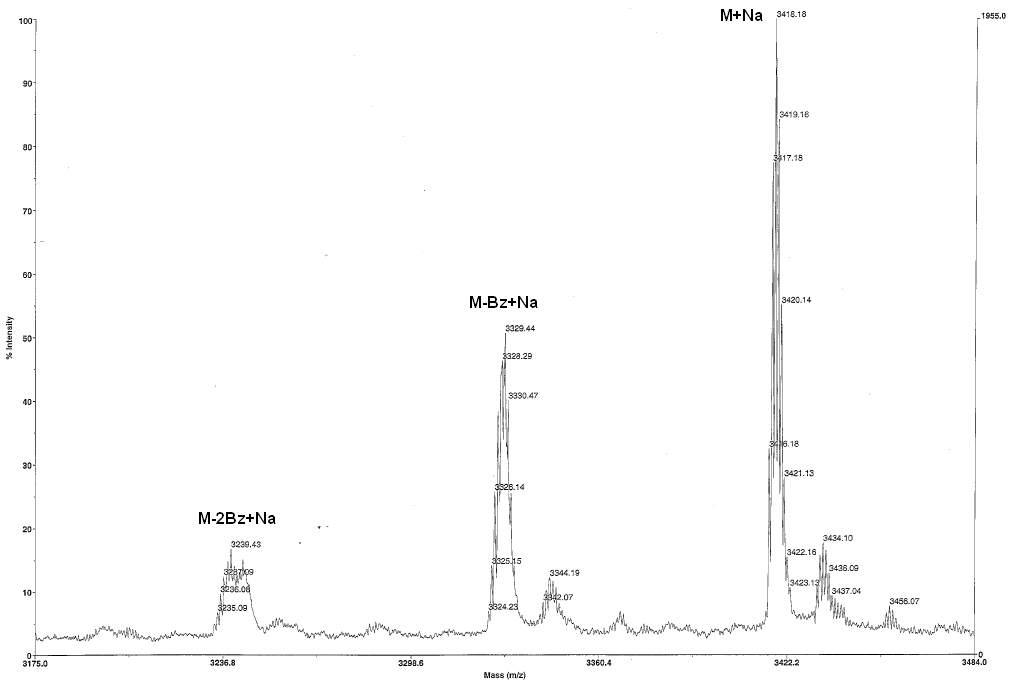


**M+Na-Bn**

**M+Na-2Bn**

**Supplementary Figure 5: MALDI-MS analysis of fraction P4 (*p*-(benzyloxy)calix[16]arene)**

**Supplementary Figure 6:  ^1^H NMR (DMSO-D_6_) analysis of fraction F6b (*p*-(benzyloxy)calix[9]arene)**

 **Supplementary Figure 7:  ^1^H NMR (DMSO-D_6_) analysis of fraction P7 (*p*-(benzyloxy)calix[10]arene)**

**Supplementary Figure 8:  ^1^H NMR (DMSO-D_6_) analysis of fraction P10 (*p*-(benzyloxy)calix[12]arene)**

**Supplementary Figure 9:  ^1^H NMR (DMSO-D_6_) analysis of fraction F16 (*p*-(benzyloxy)calix[13]arene)**

**Supplementary Figure 10:  ^1^H NMR (DMSO-D_6_) analysis of fraction P4 (*p*-(benzyloxy)calix[16]arene)**

**Supplementary Note 2: NMR analyses.**

Most giant calixarenes were found to be soluble species (preferably in DMSO, regardless of their sizes), allowing for their accurate analysis by NMR spectroscopy.

A plot of the chemical shifts vs. sizes (Supplementary Figure 11) shows that asymptotic values are reached above 14 PRU (see following figure). Such a phenomenon has already be observed by Gutsche and coworkers with *p*-(^t^Bu)calix[n]arenes ^[1](#_ENREF_1)^.

**Supplementary Figure 11: evolution of the ^1^H NMR chemical shifts of the different protons of large calixarenes (red) as a function of the ring size. Asymptotic chemical shifts values shown in boxes.**

Thus, ^1^H NMR alone cannot be used for ring sizes determination above a threshold value of 14 PRU. However, it evidences the absence of end-groups commonly associated with linear oligomers.

**Supplementary Note 3: MALDI-TOF MS analyses**

MALDI MS proved to be the most suitable and general tool for the MS analysis of giant calixarenes. Molecular peaks are usually observed with good S/N ratio.

However, we observed that the sensitivity of the MALDI MS analysis rapidly decreases as the ring size of the calixarenes increases. This induces discrepancies between SEC and MALDI analyses (see below), the later underestimating sizes distributions.

This decreased sensitivity for the MALDI MS analysis of the largest calixarenes is due to: i) increasing MALDI-induced debenzylation processes; ii) increasing hydrogen/metal exchanges; iii) ion losses effects during the analysis (in the commonly used reflectron mode); iv) reduced signals from the detector during the impact of the heaviest ions due to their reduced velocity, and v) the fact that more generally, decreased sensitivities for the largest species are also commonly observed during the MALDI MS analysis of polydisperse samples of polymers ^[2](#_ENREF_2)^.

Points i) and ii) are becoming increasingly prominents as the size range of the calixarene increases, as the probability for these debenzylations / metal exchange reactions increases concomitantly. As a result, one giant calixarene usually appears as a very complex combination of signals, resulting from combinations of up to three concomitant debenzylations/hydrogen-metal exchanges. These effects split the signals associated with one single calixarene in a multiplicity of peaks, considerably decreases the S/N ratio of this specie. To suppress this effect, the MALDI MS analysis of the largest calixarenes was performed using alkylated derivatives. Systematic experiments showed that (N,N)diethyl chloroacetamide derivatives gives the best results.

To solve point iii), the analyses of the largest calixarenes were performed in the so-called linear mode. In this case, the ions produced during the laser-induced ablation of the sample directly travel towards the detector without any deviation of the trajectory of the ions during the analysis (in sharp contrast with the usual reflectron mode). The counterpart for this increased sensitivity for the largest species is a reduced resolution. This however does not prevent accurate analysis of the samples, providing that a careful calibration of the experiment is realized during the analysis.

Materials and methods

MALDI-TOF MS analyses were performed using an UltrafleXtreme mass spectrometer (Bruker Daltonics, Bremen) or a Voyager DE-sSTR mass spectrometer (AB Sciex, les Ulis France). Acquisitions were performed in reflector or linear positive ion mode. The laser intensity was set just above the ion generation threshold to obtain peaks with the highest possible signal-to-noise (S/N) ratio without significant peak broadening. The mass spectrometer was externally calibrated using PEG. All data were processed using the program FlexAnalysis (Bruker Daltonics, Bremen) or the Data Explorer software package (AB Sciex, les Ulis France).

Trans-2-[3-(4-ter-Butylphenyl)-2-propenylidene] malonitrile (DCTB) was used as the matrix for MALDI-TOF MS. Sodium, potassium and cesium trifluoroacetate salts were used as cationizing agents. All were of the highest available grade (from Sigma Aldrich Co) and used without further purification.

**Supplementary Note 4: Size Exclusion Chromatography analyses**

In order to confirm the very high size range of our giant calixarenes, the samples were also systematically analysed by Size Exclusion Chromatography (SEC). This technique is indeed commonly used to determine the molecular weight of macromolecules ^[3](#_ENREF_3)^.

Briefly, a solution containing the molecules to be analyzed is flowed through a porous adsorbent. The smallest molecules will diffuse more efficiently into all the pores, thus increasing their retention time on the column, i.e. slowing down their displacement. On the opposite, larger ones will not be able to diffuse into the smallest porosity, and will thus elute more rapidly.

The calibration of the SEC system was done using the previously described reference samples of pure *p*-(benzyloxy)calix[n]arenes of various sizes.

The SEC analysis of these reference samples, along with the corresponding calibration curve is shown on Supplementary Figure 12.

**Supplementary Figure 12: Example of calibration curve obtained from the plotting of the molecular weight of reference samples of pure *p*-(benzyloxy)calixarenes as a function of their molecular weight**

Note that these calibration curves were systematically repeated before each analysis of giant calixarenes, in order to suppress artifacts (that may be related for example to changes in the structure of the stationary phase of the SEC column) between consecutive analyses. The molecular weight of giant calixarenes was determined using this calibration curve, from the measured retention time. Supplementary Figure 13 shows a typical example of a giant calixarene analysis by SEC. Detailed characterisations of this sample are provided p.93 of the Supplementary Informations).

**Supplementary Figure 13: Size Exclusion Chromatography analysis of a sample of giant calixarenes, obtained using the two-steps process, with a CsOH/phenol ration of 0.85.**

The PRU number, as determined from the on peak molecular weight Mp, is shown on the chromatogram**.**

The top curve shows the chromatogram of the analysed sample, the bottom one the chromatogram of a reference *p*-(benzyloxy)calix[16]arene. Qualitatively, a comparison between these two chromatograms immediately shows that the calixarenes in the analysed sample have a smaller elution volume, corresponding to a much larger size than the reference one. Quantitatively, the average molecular weight of the analysed sample was determined from the previous calibration curve (Supplementary Figure 12). In the field of macromolecular chemistry, this average molecular weight can be expressed in three different ways: the average molecular weight in number (Mn), the average molecular weight in weight (Mw), or the average molecular weight at the top of the most intense peak (on peak molecular weight, Mp). This later value is the one used all over the paper, as being simpler to handle, and corresponding to the most abundant specie in the sample. In the example shown on Supplementary Figure 13, the average molecular weight Mp determined from the on peak retention volume is 8200 g.mol-1, corresponding to a number of phenolic units for the most abundant specie of 39.

**Supplementary Note 5: Compared Size Exclusion Chromatography analysis of giant calixarenes using linear polymers and calixarenes as calibrants.**

A compared molecular weight determination was undertaken using both the previously introduced calixarenes (Supplementary Note 1, Supplementary Figure 12) and linear oligomers (PMMA, (poly)methylmethacrylate) as standards. We show on Supplementary Figure 14 an example of a calibration curve obtained with PMMA calibrants of known molecular weights.

**Supplementary Figure 14: example of calibration curve obtained using reference (poly)methylmetacrylate (PMMA) samples of known molecular weights.**

Note that the calibration curves (with both PMMA and calixarenes references) were systematically repeated before each analysis of giant calixarenes, in order to suppress artifacts (that may be related for example to changes in the structure of the stationary phase of the SEC column) between consecutive analyses.

The results of these compared experiments are shown in Supplementary Table 1.

| **Experiment** | **Results obtained from PMMA Calibration Curve** | **Results obtained from Calixarene Calibration Curve** | **PRUs number (from Calixarene calibration)** | $\frac{Mp(PMMA)}{Mp(calixarene)}$ |
| --- | --- | --- | --- | --- |
| CsOH 0.4 equivalent  Two steps,  Second precipitate  (S16E) | *M*_n_ = 7800  *M*_w_ = 12000  ***M*_p_ = 8500**  *Đ* = 1.54 | *M*_n_ = 3500  *M*_w_ = 5800  ***M*_p_ = 3500**  *Đ* = 1.67 | **17** | **2.43** |
| CsOH 0.7 equivalent  One step  (S8E) | *M*_n_ = 10900  *M*_w_ = 15000  ***M*_p_ = 9700**  Đ = 1.38 | *M*_n_ = 4800  *M*_w_ = 7500  ***M*_p_ = 4200**  *Đ* = 1.54 | **20** | **2.31** |
| RbOH 0.8 equivalent  One step  (S6G) | *M*_n_ = 8700  *M*_w_ = 12600  ***M*_p_ = 12000**  *Đ* = 1.45 | *M*_n_ = 4000  *M*_w_ = 8200  **M_p_ = 6200**  *Đ* = 2.04 | **29** | **1,94** |
| CsOH 0.8 equivalent  One step  (S9H) | *M*_n_ = 12500  *M*_w_ = 29400  ***M*_p_ = 18900**  *Đ* = 1.63 | *M*_n_ = 5500  *M*_w_ = 10200  ***M*_p_ = 8800**  *Đ* = 1.86 | **42** | **2.15** |
| RbOH 0.8 equivalent  Two steps,  Second precipitate  (S18E) | *M*_n_ = 11900  *M*_w_ = 10700  ***M*_p_ = 19500**  *Đ* = 1.61 | *M*_n_ = 5800  *M*_w_ = 10700  ***M*_p_ = 10500**  *Đ* = 1.85 | **50** | **1.86** |
| RbOH 0.8 equivalent  Two steps,  First precipitate  (S18K) | *M*_n_ = 19400  *M*_w_ = 30000  ***M*_p_ = 30900**  *Đ* = 1.55 | *M*_n_ = 9700  *M*_w_ = 18500  ***M*_p_ = 18300**  *Đ* = 1.90 | **86** | **1.7** |

**Supplementary Table 1: comparison of the molecular weights determined using PMMA and calixarenes calibrations**

(the references of the corresponding experiments in the SI are shown in parenthesis).

Note that in this table, we highlighted the on peak molecular weight values (bold), that are the ones considered in the manuscript.

We observe that the PMMA-determined on peak molecular weights of giant calixarenes are quite different from the actual ones, determined using the calixarenes calibration (calibration whose accuracy was independently confirmed by combined SEC-MALS and DLS experiments). This is not surprising, as not only linear and cyclic oligomers have very different hydrodynamic characteristics but also the thermodynamic parameters of DMF is different for PMMA or giant calixarenes, leading to different hydrodynamic volumes.

More precisely, this comparison shows that the molecular weight of giant calixarenes is overestimated by a factor of two using the PMMA calibration.

We also observe that this factor of two is quite general, and applies as well over a large molecular weight range.

Supplementary Figure 15 shows a compared SEC analysis of samples of pure, reference calixarenes (number of phenolic sub-units shown in the boxes) and reference PMMA samples. The molecular weight of the reference calixarenes is again overestimated by the PMMA calibration.

**Supplementary Figure 15: compared SEC study of reference calixarene samples and reference PMMA ones.**

Supplementary Figure 16 shows the evolution of the Mp(PMMA)/Mp(calixarene) ratio of giant calixarenes as a function of the molecular weight determined by the PMMA calibration.

**Supplementary Figure 16: evolution of the Mp(calixarene)/Mp(PMMA) ratio as a function of Mp(PMMA).**

Such a graph will be useful to get a more accurate value of the correction factor to be applied.

In conclusion, despites the fact that the PMMA calibration cannot be directly used to obtain accurate molecular weight determinations, it can indeed be used as a simple tool to get an approximate one, providing that a correction factor of about ½ is applied.

Materials and methods

The SEC characterization of the giant calixarenes was performed on a PL GPC 120 apparatus equipped with an autosapler and a RI detector, thermostated at 70°C. 2 PL Resipore columns and a precolumn were used and thermostated at 70°C. The mobile phase was a mixture of DMF and 0.01 M LiBr delivered at a flowrate of 0.7 mL min-1. The samples were prepared with a concentration of 0.25 wt% in a solution containing the mobile phase and 0.25 wt% toluene, used as flow marker. Injection volume was 20 μL.

Polymethylmethacrylate equivalent number-average, weight-average molar masses and peak maximum molar masses (Mn, Mw and Mp) and dispersities Ɖ were calculated by means of PMMA calibration curve using PMMA standards from 1.86 to 520.0 Kg.mol-1 (Agilent, USA). Calixarènes equivalent number-average, weight-average molar masses and peak maximum molar masses (Mn, Mw and Mp) and dispersities Ɖ were calculated by means of calixarenes calibration curve using calixarenes standards from 1.272 to 3.393 Kg.mol-1, obtained by the authors after precise purification and characterization (1H NMR, MALDI-MS, SEC-MALS).

**Supplementary Note 6: PGSE NMR analyses**

Pulsed Gradient NMR analysis has also been used to confirm the previously determined molecular weights. This experiment is based on the determination of the diffusion coefficients D of the samples, which are directly related to their molecular weight M (Supplementary Table 2, using reference samples of calixarenes with known sizes). From this, a calibration curve ln(D) = f[ln(M)] is plotted (see below, Supplementary Figure 17) A reasonably good agreement between PGSE analyses and SEC/DLS/SEC-MALS ones is observed. Some discrepancies that are observed may be explained by: i) the polydispersity of the samples (as giant calixarenes are obtained as a mixture of different sizes) and ii) the fact that the size range we observed expands beyond the calibration curve.

| PRUs  of reference calixarenes | Molecular weight of reference calixarenes (Da) | P30 (ms) | *D*_exp_ (m^2^ s^-1^) | *D*_theoretical_ (m^2^ s^-1^) | Error *(%)* |
| --- | --- | --- | --- | --- | --- |
| 4 | 848 | 1.05 | 1.510 10^-10^ | 1.494 10^-10^ | -1.0 |
| 5 | 1060 | 1.15 | 1.353 10^-10^ | 1.342 10^-10^ | -0.8 |
| 6 | 1272 | 1.20 | 1.227 10^-10^ | 1.229 10^-10^ | +0.1 |
| 7 | 1484 | 1.30 | 1.131 10^-10^ | 1.141 10^-10^ | +0.9 |
| 8 | 1696 | 1.35 | 1.068 10^-10^ | 1.070 10^-10^ | +0.2 |
| 9 | 1908 | 1.35 | 1.007 10^-10^ | 1.010 10^-10^ | +0.3 |
| 10 | 2120 | 1.40 | 9.585 10^-11^ | 9.603 10^-11^ | +0.2 |
| 12 | 2544 | 1.5 | 8.841 10^-11^ | 8.795 10^-11^ | -0.5 |
| 16 | 3392 | 1.55 | 7.743 10^-11^ | 7.655 10^-11^ | -1.1 |

**Supplementary Table 2: experimental and theoretical diffusion coefficients D for reference samples of *p*-(benzyloxy)calixarenes of various molecular weights (PRUs: Phenolic Repetition Units).**

**Supplementary Figure 17: Calibration curve obtained from the previously determined diffusion coefficients**

Materials and methods

The experiments were performed at 20°C on a Bruker AMX 500 500MHz spectrometer, using DMSO as the solvent.

**Supplementary Note 7: Size Exclusion Chromatography-Multiple Angle Light Scattering analyses**

The previously mentioned SEC and PGSE analyses give reliable molecular weight (MW) determination, providing that an accurate calibration curve is available. Despite the fact that this reference curve is obtained using pure calix[n]arenes as references, the observed discrepancies between SEC/PGSE and MALDI prompted us to find another technique for molecular MW determination, that wouldn’t suffer from uncertainties related to the need for calibration curve. The SEC-MALS analysis allows for such an absolute MW determination to be obtained, without any calibration curve (^[[1]](#footnote-4)^).

The working principle of a MALS detector is shown on Supplementary Figure 18.

**Supplementary Figure 18: working principle of the MALS experiment.**

The products flowing out of a conventional SEC column are sent into the MALS detector. The scattered light from the incident laser beam is collected at different angles using different detectors. This allows for absolute molecular weight determinations using the Zimm equation ^[4](#_ENREF_4)^. This determination relies on the fact that the intensity of the scattered light by a given object is proportional to the product of the molecular weight of the scattering object by its concentration. The only input parameter is the dn/dc coefficient, linking the change of the refractive index of the solution as a function of the concentration of the analysed solute. A typical example of a SEC-MALS analysis of a purified giant calixarenes sample is shown on Supplementary Figure 19. Detailed characterisations of this sample are provided p.53.

**Supplementary Figure 19: Example of SEC-MALS analysis of a giant calixarene sample (PRU n number shown in the box).**

The different expressions of the average molecular weight (Mn, Mw and Mp) were already discussed (see Supplementary Note 4, Size Exclusion Chromatography). Here, the on peak average molecular weight (Mp) was used. The experimental results are obtained as two curves. The first one shows the elution of the product out of the SEC column as detected by the refractive index change (Supplementary Figure 19, RI, blue chromatogram). The second one is the detection of the products by the Dynamic light scattering phenomenon (DLS, red curve). A third grey curve is added, showing the molecular weight determination of the eluted products, as determined from the DLS signal. The useful signal for the molecular weight determination is the light scattering one (red). However, the light scattering effect scales as R^6^ (R: radius of the molecules in solution). It thus considerably overestimates the amount of the largest species in solution. It is thus necessary to use the RI curve (blue) to determine the elution volume of the most abundant species in the analyzed sample. Indeed, the refractive index signal do not suffer from this size dependence. From this volume (green dashed line), it is possible to determine the absolute molecular weight from its intercept with the grey curve (green arrow).

As shown below, a good correspondence between SEC and SEC-MALS is observed for the samples analyzed, making us confident in the validity of the sizes of giant calixarenes we determined.

In order to check the accuracy of the SEC-MALS analysis of calixarenes, a test experiment was performed using a reference sample of *p*-(benzyloxy)calix[16]arene. The result are shown on the following chromatogram (Supplementary Figure 20).

**Supplementary Figure 20: Example of SEC-MALS analysis of a reference calix[16]arene sample.**

dn/dc = 0.14

We recall here that the results of a SEC-MALS analysis are obtained as two chromatograms, one obtained using a light scattering (LS) detector (red curve), the second one using a refractometric one (blue curve).

The measured value is 3365 g/mol, in very good agreement with the theoretical one (3393 g/mol).

As explained above, this result is an absolute molecular weight determination, that was obtained without any calibrant. The SEC-MALS analysis is thus free from the different artifacts that may be associated with any calibration-based molecular weight determination.

**Supplementary Figure 21: Example of SEC-MALS analysis of a functionalised reference calix[8]arene sample.**

dn/dc = 0.185

Mp = 2860 g/mol.

Supplementary Figure 21 shows the SEC-MALS analysis of an *N,N-*(diethyl)acetamide functionalised *p*-(benzyloxy)calix[8]arene. One again, a good agreement is found between the theoretical (2602 g/mol) and experimentally determined (2860 g/mol) molecular weights (taking into account the fact that the accuracy of the SEC-MALS analysis is lower for low molecular weight compounds).

Once again, this experiment was realized without any calibration, and is thus an absolute molecular weight determination.

The results obtained using the two previous reference samples demonstrates the reliability of the others SEC-MALS analyses that we provide.

In order to check the accuracy of the SEC / SEC-MALS molecular weight determinations, a chromatographic purification of the giant calixarene sample shown on Supplementary Figure 19 was undertaken by conventional column chromatography (SiO_2_, eluent gradient CH_2_Cl_2_ 100% 🡪 CH_2_Cl_2_ / EtOAc 2%, see also p. 62 for further details).

This chromatographic work allowed us to obtain a fraction with a SEC-determined molecular weight distribution (Supplementary Figure 22A, red chromatogram) centered at the same value (green vertical line) than the “on peak” one observed for the starting sample (Supplementary Figure 22A, black chromatogram).

**Supplementary Figure 22: compared SEC / SEC MALS and MALDI analyses.**

A MALDI-MS analysis of the former chromatographic fraction (Supplementary Figure 22A, red chromatogram) shows that it is composed of a reduced number of giant calixarenes, exhibiting a distribution around 25-26 PRUs (Supplementary Figure 22B). This range is close to the one obtained using the previously described SEC-MALS analysis (28, Supplementary Figure 19). This correspondence between the molecular weights determined by SEC/SEC-MALS on one hand and MALDI MS on the other hand confirms the validity of our approach for molecular weights determinations.

Materials and methods

The SEC-MALS analyses were performed using thermostated columns at 50°C. Two columns were used: Tosoh Alpha 2500 (exclusion limit: 5 000) + Tosoh Alpha 3000 (exclusion limit: 90 000). The eluent used was a 10 mM LiBr in DMF solution, at a 1 mL/min flow rate. The injected volume was 50μL.

The detection was made using two detectors:

- Differential refractometric detector: Optilab Rex, Wyatt;
- Multiangles static light diffusion detector: TREOS Wyatt, 3 angles, laser wavelength = 658 nm.

For the interpretation of the results, the ASTRA VII (Wyatt Technology) software was used.

Alternatively, the SEC-MALS analyses were performed using a MALVERN “VISCOTEC” SEC-MALS 20 apparatus, in THF solutions, using a 35°C thermostated column.

**Supplementary Note 8: Dynamic Light Scattering analyses**

Dynamic Light Scattering (DLS) allows for the determination of the hydrodynamic radius of macromolecular objects by observing their diffusion of a monochromatic laser light in solution. Indeed, the intensity of the diffused light scales as R_h_^6^ (R_h_: hydrodynamic radius). For the smallest members of the giant calixarene family (number of PRUs ≤ 30), the sizes measured by DLS are in good agreement with their theoretical diameter (Supplementary Figure 23) and with SEC results. This theoretical diameter is obtained from a simple calculation. Given the diameter of a reference calix[8]arene (1.3 nm, molecular modelling), the length ***L*** of the repeating unit (-Ar-CH_2_-) is 0.51nm. Using the formula ***P = πD = nL*** (***P*** = Perimeter, ***n*** = number of repeating units), the number of repeating units is directly deduced from the DLS-determined diameter as ***n = πD/L***.

Supplementary Figure 23 shows examples of such calculations:

**Supplementary Figure 23: the geometric model used for the size determination of calixarenes**

This good correlation between DLS/SEC on one hand and the theoretical diameter of giant calixarenes on the other hand may appear surprising at first sight. Indeed, NMR shows giant calixarenes to be highly fluxional objects. This shows that from the hydrodynamic point of view (and within the previously mentioned size range), the macromolecular chain of giant calixarenes is very fluxional, and explores the whole volume included into a sphere with a diameter corresponding to the fully unfolded calixarene (Supplementary Figure 24A). For the largest calixarenes, the simple model shown on Supplementary Figure 23 is no longer valid, as the DLS-observed sizes are smaller than expected, considering the molecular weights obtained by SEC and SEC-MALS. This could be explained first by considering the hypothesis used for the analysis of experimental diffusion data (calixarenes considered as equivalent to polystyrene latexes nanoparticles). Moreover, the largest calixarenes may adopt a more densely packed organisation, with multiply folded conformations (Supplementary Figure 24B) and consequently a reduced apparent hydrodynamic diameter for a given molecular weight. Indeed, the probability for such folding to occurs increases with size. This is likely to result in smaller apparent hydrodynamic diameter. However, even for the largest calixarenes, a qualitative agreement is still observed between DLS and SEC/SEC-MALS, the largest calixarenes giving the largest DLS-observed sizes.

**Supplementary Figure 24: Evolution of the conformation of giant calixarenes as a function of their diameter**

Materials and methods

The solutions for DLS analyses were prepared by dissolving 15 mg of the calixarene sample in 2 mL of DMSO. The solutions were then stirred for 10 min., filtered (0.2µ filter), and analysed. No differences were found in the observed sizes distributions upon repeating the measurement on the same solution 24h later. The analyses were performed on a Malvern Nano-ZS zetasizer, operating at 633nm.

**Supplementary Note 9: Study of the evolution of the hydrodynamic radius of giant calixarenes as a function of their molecular weight: scale law determination.**

In dilute solutions, the diffusion coefficient D of a macromolecular object (polymer) is related to its hydrodynamic radius R_h_ by the Stokes-Einstein equation (Supplementary Equation 1):

$D=\frac{kT}{6\eta\pi Rh} \sim\frac{K}{Rh}$

**Supplementary Equation 1**

(k; Boltzman’s constant; T: temperature, η: viscosity of the solvent, Rh: hydrodynamic radius)

This can also be expressed as a function of the molecular weight M of the analysed molecule ^[5](#_ENREF_5)^, using the following power law (Supplementary Equation 2):

${\frac{K}{Rh}=M}^{-\nu} {\leftrightarrow Rh\sim M}^{\nu}$

**Supplementary Equation 2**

The coefficient **ν** is an exponent characteristic of the hydrodynamic behavior of the polymeric chains. This may be summarized as (Supplementary Equations 3 and 4):

${Rh\sim M}^{\nu}$

**Supplementary Equation 3**

${D\sim M}^{-\nu}$

**Supplementary Equation 3**

From the data shown on Supplementary Table 2 (log of the diffusion coefficient D vs. the log of the molecular weight of reference calixarene samples), the corresponding scale law was plotted. The result is shown on Supplementary Figure 25.

**Supplementary Figure 25: Evolution of the logarithm of diffusion coefficient of reference samples of calixarenes as a function of the logarithm of their molecular weight.**

As expected from the previous equation, a linear relationship is found, with a slope of -0.48. In the same way, Supplementary Figure 26 shows the plot of the scale law linking the logarithm of the hydrodynamic diameter (as determined by DLS) with the logarithm of the molecular weight of giant calixarenes. The numbers in boxes are the number of phenolic repetition units of the corresponding giant calixarenes (as determined from the “on peak” sizes Mp of the SEC chromatograms).

**Supplementary Figure 26: Evolution of the logarithm of the hydrodynamic diameter (Dh) of samples of giant calixarenes as a function of the logarithm of their molecular weight Mp.**

A linear relationship is found, with a slope of +0.49. The absolute value of this slope is close to the one found on the previous graph (0.48). This is not surprising, as the diffusion coefficient D (measured by ^1^H NMR) and the hydrodynamic radius (measured by DLS) are both directly related to M by the same exponent **ν** (from the De Gennes’s equation). The fact that the same exponent is found for both reference calixarenic samples and giant calixarenes confirms that they all have the same hydrodynamic behavior. This can only be the case if both compounds have the same cyclic structure. More precisely, an exponent close to 0.5 is characteristic of a random-coil behavior in theta solvent, without chain expansion (due to the cyclic structure of giant calixarenes). Such a coefficient is consistent with the high flexibility expected for giant calixarenes.

**Supplementary methods**

**General**

All the reagents and solvents were obtained from TCI, and used without any further purification. ^1^H and ^13^H NMR were recorded on Bruker AC 250, 300 and 360 MHz. All the spectra were recorded in DMSO (D6.)

**Giant calixarenes: one-step process**

**Synthesis and purification**

**Synthesis:**

In a typical synthesis, a suspension of 100g of *p*-(benzyloxy)phenol and 15g of paraformadehyde in 700 mL of xylene (technical grade) is loaded under argon in a 2-L, three necked flask fitted with a Dean-Stark collector, a magnetic stirrer and a heating oil bath. The system is then flushed with argon under strong stirring and kept under argon all the synthesis long. The heating bath is then switched on. At 90°C, the required amount of base (from 0.3 to 0.85 equivalent vs. phenol) is then added as a 50% solution (w/w) in water. The suspension is then refluxed for 6 hours (0.3 equivalent of base) to 15 hours (0.8 equivalent of base).

**Purification:**

Purification process A: DMSO/acetone recrystallization.

After cooling the reaction media down to ambient temperature, 500mL of THF are added, and the suspension is neutralized by slowly adding 1.1 equivalent (vs. initially introduced base) of 37% HCl under strong stirring. The reaction media is then evaporated to dryness. The obtained solid is then suspended in 2L of methanol under strong stirring for two days and filtered. The cream-colored solid is then suspended in 2L of acetonitrile under strong stirring for 2 days and filtered. After drying, the precipitate is dissolved in 120 mL of DMSO at 100°C. 2L of acetone are then rapidly added, and the resulting suspension is hot filtered, leaving a precipitate of *p*-(benzyloxy)calix[8]arene (combined with giant calixarenes in some cases, especially if RbOH is used). The corresponding filtrate is stored for 3 days at 1°C, leaving a microcrystalline precipitate. This precipitate is filtered, washed with a DMSO/acetone solution (10/90 v/v), then pure acetone, and dried under vacuum. Analyses show this precipitate to be only constituted of giant calixarenes, as a mixture of different ring sizes.

**Supplementary Figure 27: purification process A: acetone-DMSO crystallization of giant calixarenes.**

Purification process B: DMSO/EtOH recrystallization

120 g of neutralized, methanol washed crude product were dissolved in 150 mL of DMSO. 150ml of ethanol are then added. This solution is stored overnight at 1°C. The resulting precipitate is collected by filtration, and dried under vacuum. Analyses show this precipitate to be only constituted of giant calixarenes, as a mixture of different ring sizes.

Note: These syntheses were upscaled up to 400g of starting *p*-(benzyloxy)phenol without any difference in the composition/yield.

**Characterisations:**

MALDI-MS: see Supplementary Note 3 for a detailed description of the MALDI-TOF-MS analysis of giant calixarenes.

NMR:

^1^H NMR (250 MHz, DMSO, 20°C): δ (ppm) = 8.36 (OH); 7.5-7 (m, 5H, ArH); 6.52 (s, 2H, Ar_(hydroquinone)_H; 4.77 (s, 2H, ArCH_2_O); 3,84 (br. s, 2H, ArCH_2_Ar).

^13^C NMR (100 MHz, DMSO, 20°C): δ (ppm) = 31.32 (Ar-CH2-Ar); 69.77 (OCH_2_Benzyl); 114.65 (Ar_(hydroquinone)_C-H); 126-130 (ArC-H); 137.76 (Ar_(hydroquinone)_C*ipso*-CH_2_); 140,24 (ArC*ipso*-CH_2_); 152.11 ((Ar_(hydroquinone)_C*ipso*-OH).

**One-step process: RbOH-catalysed reaction**

**RbOH 0.3 equivalent vs. phenol**

**Supplementary Figure 28: crude product -** ^1^H NMR (DMSO-d6). Bottom: zoom

**Supplementary Figure 29: purified product** **-** ().

Yield of purified giant calixarenes: 15% (purified using DMSO/acetone recrystallization).

**Supplementary Figure 30: purified product - MALDI MS analysis** (cationised using CF_3_COO-Na+).

**RbOH 0.6 equivalent vs. phenol**

**Supplementary Figure 31: crude product –** ^1^H NMR (DMSO-d6); red asterisk : residual xylenes).

**Supplementary Figure 32: crude product – MALDI-MS analysis** (cationised by CF_3_COOK). A) global; B) zoom.

pp

**Supplementary Figure 33: crude product – SEC analysis. Top: global; bottom: zoom.**

PRUs shown in boxes.

**Supplementary Figure 34: purified product –** ().

Yield of purified giant calixarenes: 20% (purified using process A).

**Supplementary Figure 35: purified product - ^13^C NMR** (DMSO-d6).

**Supplementary Figure 36: purified product - MALDI MS** (after CF_3_COONa addition)

Zoom highlighting photoinduced debenzylations (M-91) and H+/Na+ exchanges (M+22):

**Supplementary Figure 37: purified product - Size Exclusion Chromatography**

PRU number shown in the box.

**Supplementary Figure 38: purified product - Size Exclusion Chromatography-Multiple Angle Light Scattering (SEC-MALS).** PRUs number is shown in the box.

dn/dc = 0.17

**RbOH 0.85 equivalent vs. phenol**

**Supplementary Figure 39: crude product - ^1^H NMR** (DMSO-d6). A): global; B): zoom

**Supplementary Figure 40: crude product – SEC**. PRU number shown on chromatogram. Reference: *p*-benzyoxycalix[16]arene.

**Supplementary Figure 41: crude product - MALDI MS** (after addition of CF_3_COOCs).

pp

**Supplementary Figure 42: purified product-**()**.**

Yield of purified giant calixarenes: 61% (purified using the DMSO/EtOH recrystallization process).

**Supplementary Figure 43: purified product -  ^13^C NMR** (DMSO-d6).

**Supplementary Figure 44: purified product - MALDI MS** (after addition of CF_3_COOCs). Top: global; bottom: zoom

PRUs numbers are shown on spectra.

**Supplementary Figure 45: purified product - Size Exclusion Chromatography (SEC)**

PRU number shown on chromatogram.

**Supplementary Figure 46: purified product - DLS**

**Purified product - PGSE NMR analysis**

D = 0.56, M = 4760

**Supplementary Figure 47: purified product - Size Exclusion Chromatography-Multiple Angle Light Scattering analysis.**

PRUs number is shown in the box.

**Supplementary Figure 48: purified product - Size Exclusion Chromatography-Multiple Angle Light Scattering analysis of an acetamide-derivatised sample.**

PRUs number is shown in the box.

The molecular weight value determined here for a derivatized sample is coherent with the one determined from for the parent, unfunctionalised calixarene (see Supplementary Figure 47).

**Purified product - combined SEC and MALDI-MS analyses of giant calixarenes.**

A sample of the previously discussed purified giant calixarenes was subjected to an extensive chromatographic partitioning. (SiO_2_, eluent gradient: CH_2_Cl_2_ 100%🡪 CH_2_Cl_2_ 98% / EtOAc 2%). The different fractions obtained were then washed with ethyl acetate. Both the ethyl acetate filtrates and precipitates were separately analysed by SEC.

The SEC analysis of the most interesting fraction is shown on the following figure.

This fraction shows a size range (red chromatogram, supplementary Figure 49A) centered on the on peak value (shown as a green vertical line) of the starting calixarenes mixture (Supplementary Figure 49A black chromatogram). This means that this chromatographic fraction contains the most abundant species present in the starting calixarenes mixture.

**Supplementary Figure 49: SEC (A) and MALDI MS (B) analyses of a chromatographic fraction of giant calixarenes (PRUs shown in the boxes)**

Supplementary Figure 49B shows a comparition between the previous chromatographic fraction (red chromatogram) and a diluted solution of the starting calixarenes mixture (black chromatogram). There is no difference between the observed elution volumes. This rules out the possibility for artifacts (i.e. changes in the apparent elution volume) arising from concentration differences between the two samples.

A MALDI-MS analysis of this chromatographic fraction showed it to be composed of a narrowed range of calixarenes (Supplementary Figure 49C), with a size distribution centered on 25-26 PRUs. This value is close from the ones obtained by SEC (29 PRUs) and SEC-MALS (28 PRUs).

***p*-(benzyloxy)calix[25]arene.**

This calixarene (1 mg) was obtained from the previous chromatographic fraction (red chromatogram, Supplementary Figures 22, 49A and 49B) by preparative TLC (SiO_2_, eluent; CH_2_Cl_2_/EtOAc 99.5/0.5 v/v; Rf=0.5).

**^1^H Supplementary Figure 50: ^1^H NMR** (DMSO-d6).

**Supplementary Figure 51: ^13^C NMR** (DMSO)D_6_

**Supplementary Figure 52: MALDI MS analysis.**

**One-step process: CsOH-catalysed reaction**

**CsOH 0.3 equivalent vs. phenol**

**Supplementary Figure 53: crude product - ^1^H NMR** (DMSO-d6)

**Supplementary Figure 54: purified product – ^1^H NMR** (DMSO-d6):

Yield of purified giant calixarenes: 10 % (purified using process A).

**Supplementary Figure 55: purified product - ^13^C NMR** (DMSO-d6)**:**

**Supplementary Figure 56: purified product - MALDI MS**

PRU number and cations shown in boxes.

**CsOH 0.7 equivalent vs. phenol**

**Supplementary Figure 57: crude product - ^1^H NMR** (DMSO-d6):

**Supplementary Figure 58: purified product – ^1^H NMR** (DMSO-d6):

Yield of purified giant calixarenes: 20% (purified using process A).

**Supplementary Figure 59: purified product - ^13^C NMR** (DMSO-d6);

**Supplementary Figure 60: purified product - MALDI MS (after addition of CF_3_COOK).** A) global; B) photoinduced debenzylations and C) hydrogen/metals exchanges.

PRUs numbers are shown on spectra.

**Supplementary Figure 61: purified product - SEC**

PRU number shown on chromatogram.

Mp = p4200 g/mol, Mw = 7500 g/mol, Mn = 4800g/mol, PDI: 1.54.

**Supplementary Figure 62: purified product – DLS**

**CsOH 0.85 equivalent vs. phenol**

**Supplementary Figure 63: crude Product - ^1^H NMR** (DMSO-d6). Bottom: zoom.

Note: green asterisks = bis(homooxa) *p*-(benzyloxy)calix[4]arene ^[6](#_ENREF_6)^.

**Supplementary Figure 64: crude product - MALDI MS** (after addition of CF_3_COOK). A) global; B) zoom

pp

Note: Red asterisks: photoinduced debenzylations; Green asterisks: hydrogen/metal exchanges.

**Supplementary Figure 65: crude product - Size Exclusion Chromatography (SEC) analysis**

PRUs number is shown in the box.

Mp = 9200 g/mol, Mw = 7300g/mol, Mn = 2800g/mol.

**Crude product – SEC-MALS analyses of Functionalised derivatives.**

1. Synthesis of an acetylated derivative:

To an ice-cooled solution of 0.6g of crude giant calixarenes and 2 ml of triethylamine in 10 ml of THF are added dropwise 2 ml of acetic anhydride.

The resulting suspension is stirred overnight at ambient temperature. The suspension is filtered, and the filtrate evaporated to dryness.

The resulting solid is washed with water, and dried under vacuum.

1. Synthesis of a triflated derivative:

To an ice-cooled solution of 0.6g of crude giant calixarenes and 2 ml of triethylamine in 10 ml of THF are added dropwise 0.5 ml of (trifloromethanesulfonyl)chloride.

The resulting suspension is stirred overnight at ambient temperature. The resulting suspension is extracted with dichloromethane and water. The organic phase is washed with a saturated NaHCO_3_ solution, dried over sodium sulfate and evaporated.

The resulting solid is dried under vacuum.

1. Analyses:

**Supplementary Figure 66: SEC-MALS analyses of functionalised samples.**

The two SEC-MALS analyses of both acetylated and triflated derivatives gave coherent molecular weight values, that are in accordance with the ones obtained by SEC analysis. The later is higher than the former due to the increased molecular weight of the triflated repeating unit compared with the acetylated one.

**Supplementary Figure 67: purified product – ^1^H NMR** (DMSO-d6):

Yield of purified giant calixarenes: 65% (purified using process A: DMSO/acetone recrystallization).

**Supplementary Figure 68: purified product - ^13^C NMR** (DMSO-d6):

**Supplementary Figure 69: purified product - MALDI MS** (after addition of CF_3_COOCs)

PRU numbers shown in boxes.

pp

**Supplementary Figure 70: purified product - Size Exclusion Chromatography (SEC)**

PRU number shown on chromatogram.

Mp = 8800 g/mol, Mw = 10200g/mol, Mn = 5500g/mol.

**Supplementary Figure 71: purified product – DLS**

**Purified product - PGSE NMR analysis**

D=0.56, M=6500 g/mol.

**Effect of RbOH concentration on both the yield and size distribution of giant calixarenes (one-step process, crude products)**

**Supplementary Figure 72: Ccompared ^1^H NMR (DMSO-d6), MALDI-TOF and SEC analysis of crude samples of two experiments run with 0.6 and 0.8 equivalent of RbOH respectively. MALDI-TOF MS analyses shown on figures F and G performed in the linear mode, using CF_3_COOCs as the cationising agent. PRU numbers shown in boxes.**

**Effect of RbOH concentration on both the yield and size distribution of giant calixarenes (one-step process, purified products)**

**Supplementary Figure 73: compared ^1^H NMR (A, D; DMSO-d6), MALDI-TOF (B, E) and SEC (C, F) analyses of purified samples of two experiments run with 0.6 equivalent (20% yield) and 0.85 equivalent (61% yield) of RbOH respectively.**

The MALDI-TOF MS analyses shown on figures B and E were performed in the linear mode, using CF_3_COONa and CF_3_COOCs as the cationising agent, respectively.

Note:

- Giant calixarenes from the RbOH 0.85 equivalent synthesis were purified using process B (DMSO/EtOH precipitation);

- Giant calixarenes from the RbOH 0.6 equivalent synthesis were purified using process A (DMSO/acetone precipitation).

The PRU numbers are shown in boxes.

**Effect of CsOH concentration on both the yield and size distribution of giant calixarenes (one-step process, purified products)**

 **Supplementary Figure 74: compared ^1^H NMR (A, B; (DMSO-d6), MALDI-TOF (C, D) and SEC (E🡪H) analyses of purified samples of two experiments run with 0.6 equivalent (20% yield) and 0.85 equivalent (65% yield) of CsOH respectively. The MALDI-TOF MS analyses shown on figures C and D were performed in the reflectron and linear mode, using CF_3_COOK and CF_3_COOCs as the cationising agent, respectively.**

Note:

- Both samples purified using the acetone/DMSO purification process A.

- Comparison of the SEC chromatograms with a *p*-(benzyloxy)calix[16]arene reference sample.

- PRU numbers shown in boxes.

**Giant calixarenes: two-step process**

**Synthesis and purification**

**Synthesis:**

A 2L three necked round bottomed flask fitted with a mechanical stirrer, a Dean-Stark collector, and a heating mantle is loaded with 104g of *p*-(benzyloxy)phenol and 130mL of 37% formaldehyde. One of the lateral necks is connected via a (closed) glass valve to a water-filled bubbler. The system is then purged with argon. An aqueous 50% weight solution of base (accounting for a base/phenol ration between 0.3 and 0.85) is then added under strong argon flushing, and both the mechanical stirrer and the heating mantle are switched on. The initial thick suspension rapidly turns into a deep yellow clear solution. The reaction is then refluxed for 30 minutes. While keeping the reaction media under reflux, the lateral glass valve is then open while increasing the argon debit, thus enabling a fast argon stream (about 10 bubbles/s) trough the flask. This results in a fast removal of water, up to complete evaporation and solidification of the reaction media, resulting in a yellow to orange solid (depending on the initial base loading): the precursor. The mechanical stirrer is then switched off. The duration of this evaporation shows a surprising dependence on base: the higher the amount of base used, the lower the evaporation time (from 30 minutes to 1h, respectively). 500mL of xylene are then added, and the system is then refluxed under strong mechanical stirring for 10 hours under argon. After cooling down to ambient temperature, the resulting thick suspension is neutralized with a solution of 1.2 equivalent of HCl (37%, aq.) vs. the initial amount of base in 700mL of THF, under strong mechanical stirring for 24h. The resulting fluid suspension is then evaporated to dryness.

**Purifcation:**

Purification process A: DMSO/acetone recrystallization

The previously obtained solid is suspended in 2L of MeOH under strong stirring for 2 days. After filtration and drying, the precipitate is then washed with 2L of acetonitrile under strong stirring for 2 days. After filtration and drying, the precipitate is dissolved in 120mL of DMSO at 120°C under argon. 2L of acetone are then hot added, and the resulting suspension is hot filtered. The filtrate is then kept at 1°C for 3 days, resulting in the formation of a microcrystalline precipitate on the walls of the glassware. This precipitate is recovered by filtration, washed with an acetone/DMSO (90/10) solution, then with pure acetone and dried under vacuum.

Purification process B: DMSO/ethanol recrystallization

The crude solid obtained at the end of the second annealing step is suspended in 2L of MeOH under strong stirring for 2 days. After filtration and drying, the precipitate is then washed with 2L of acetonitrile under strong stirring for 2 days. After filtration and drying, the precipitate is dissolved in 120mL of DMSO at 120°C under argon. After cooling down to ambient temperature, 120mL of ethanol are then added, and the resulting solution is left at 1°C for three days. The resulting precipitate is then filtered, washed with EtOH and dried under vacuum.

**Supplementary Figure 75: ^1^H NMR (DMSO-d6) comparative analysis of two crude samples of giant calixarenes obtained using different base/phenol ratio (two-steps process)**

**Supplementary Figure 76: ^1^H NMR comparative analysis (DMSO-d6) of two crude samples of giant calixarenes obtained using different processes (0.8 equivalent RbOH each).**

**Two-steps process: CsOH catalysed reactions**

**CsOH 0.4 equivalent vs. phenol**

**
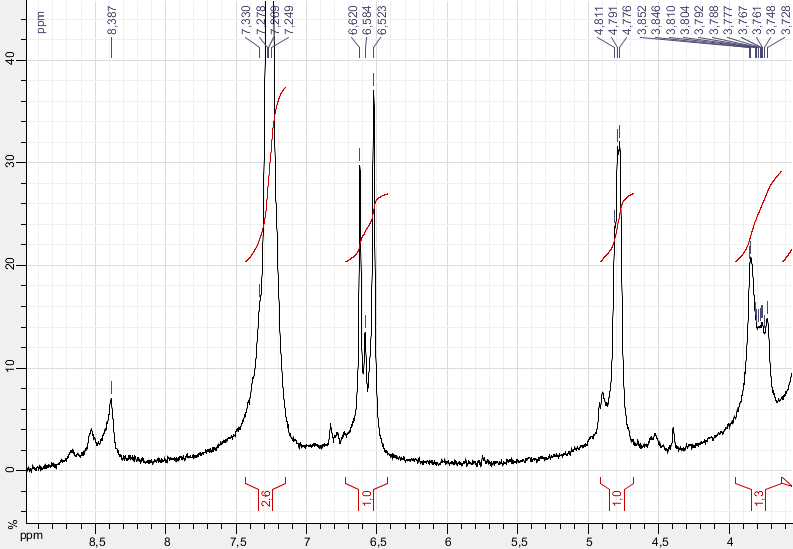
Supplementary Figure 77: crude product - ^1^H NMR** (DMSO-d6):

**Supplementary Figure 78: purified giant calixarenes – ^1^H NMR** (DMSO-d6):

Yield of purified giant calixarenes: 30% (purified using DMSO/acetone recrystallization).

**Supplementary Figure 79: purified giant calixarenes - ^13^C NMR** (DMSO-d6):

**Supplementary Figure 80: purified giant calixarenes - MALDI MS** (after addition of CF_3_COOK)

PRUs numbers are shown on the spectra.

**C16**

**Supplementary Figure 81: purified giant calixarenes - Size Exclusion Chromatography**

PRUs numbers are shown on the spectra.

Mp = 3500 g/mol, Mw = 5800 g/mol, Mn = 3500 g/mol

**Supplementary Figure 82: purified giant calixarenes - DLS (DMSO)**

**CsOH/phenol = 0.85**

**Supplementary Figure 83: crude product - ^1^H NMR** (DMSO-d6).

**Supplementary Figure 84: purified sample - ^1^H NMR** (DMSO-d6).

 **Supplementary Figure 85: purified sample - ^13^C NMR** (DMSO-d6).

**Supplementary Figure 86: purified sample - MALDI MS analysis** (after addition of CF_3_COOCs)

PRUs numbers are shown in the boxes.

See zoom on the following page:

**Supplementary Figure 87: purified sample - MALDI MS analysis** zoom

**Supplementary Figure 88: purified sample - SEC**

PRU number are shown on the chromatogram.

**Supplementary Figure 89: purified sample - DLS**

**Two-steps process: RbOH catalysed reactions**

**RbOH/phenol = 0.8**

**Supplementary Figure 90: crude product - ^1^H NMR** (DMSO-d6, Red asterisk: residual xylene).

**Supplementary Figure 91: purified giant calixarenes: ^1^H NMR** (DMSO-d6).

Yield: 50% (from 122g starting phenol, purified using the DMSO/acetone recrystallization process).

**Supplementary Figure 92: purified giant calixarenes - ^13^C NMR** (DMSO-d6).

pp

**Supplementary Figure 93: purified giant calixarenes - MALDI MS** (after addition of CF_3_COOCs).

PRU numbers shown on the spectra.

**Supplementary Figure 94: purified giant calixarenes - Size Exclusion Chromatography (SEC)**

PRUs number is shown on the chromatograms.

**Supplementary Figure 95: purified giant calixarenes - Size Exclusion chromatography-Multiple Angle Light Scattering analysis**

PRU number is shown on the chromatogram.

dn/dc = 0.14

**Purified giant calixarenes - PGSE NMR analysis**

D=0.53, M=7265 (Mn).

**Supplementary Figure 96: purified giant calixarenes - DLS**

**DMSO/acetone crystallization - hot filtration precipitate analysis**

^1^H NMR estimated yield: 14% (from 122g starting phenol).

**Supplementary Figure 97: crude products, ^1^H NMR** (DMSO-d6)

Red asterisk: calix[8]arene; green asterisk: giant calixarenes.

**Supplementary Figure 98: crude product,** **MALDI MS** (after addition of CF_3_COOCs)

PRUs numbers are shown in the boxes.

**Supplementary Figure 99: Size Exclusion Chromatography**

PRU indicated on the chromatograms.

Red asterisk: calix[8]arene; green asterisk: calix[n]arenes.

Mp = 18500 g/mol, Mn = 8500 g/mol, Mw = 18300 g/mol, Đ = 2.16.

**Supplementary Figure 100: crude products, DLS**

**Supplementary Figure 101: crude product, SEC-MALS analysis.**

The PRU number is shown in the box.

**Supplementary Figure 102: ^1^H NMR comparison of one and two-steps processes run with 0.8 equivalent RbOH vs. phenol**

PRUs are shown in boxes.

**A** and **B**: crude samples, **C 🡪 H**: purified samples.

**Solid-state synthesis**

**Synthesis**

A 2L three necked round bottomed flask fitted with a mechanical stirrer, a Dean-Stark collector, and a heating mantle is loaded with 104g of *p*-(benzyloxy)phenol and 130mL of 37% formaldehyde. One of the lateral necks is connected via a (closed) glass valve to a water-filled bubbler. The system is then purged with argon. An aqueous 50% weight solution of base (accounting for a base/phenol ratio between 0.3 and 0.85) is then added under strong argon flushing, and both the mechanical stirrer and the heating mantle are switched on. The initial thick suspension rapidly turns into a deep yellow clear solution. The reaction is then refluxed for 30 minutes. While keeping the reaction media under reflux, the lateral glass valve is then open while increasing the argon debit, thus enabling a fast argon stream (about 10 bubbles/s) through the flask. This results in a fast removal of water, up to complete evaporation and solidification of the reaction media, resulting in a yellow to orange solid (depending on the initial base loading): the precursor. The mechanical stirrer is then switched off. 300mL of heat transmitter (octane or silicone oil) are then added, and the solid material immersed in the heat transmitter is heated at 120° for 20h, while keeping the mechanical stirrer off. The reaction is monitored by taking out fragments of the solid using tweezers. After cooling down to ambient temperature, the solid material is filtered, and neutralized using 1.2 equivalent of 37% aq. HCl vs. the initial amount of base in 700mL of THF, under strong mechanical stirring for 24h. The resulting fluid suspension is then evaporated to dryness.

**Purification**

The previously obtained solid is suspended in 2L of MeOH under strong stirring for 2 days. After filtration and drying, the precipitate is then washed with 2L of acetonitrile under strong stirring for 2 days. After filtration and drying, the precipitate is dissolved in 120mL of DMSO at 120°C under argon. 2L of acetone are then hot added, and the resulting suspension is hot filtered. The filtrate is then kept at 1°C for 3 days, resulting in the formation of a microcrystalline precipitate on the walls of the glassware. This precipitate is recovered by filtration, washed with an acetone/DMSO (90/10) solution, then with pure, acetone and dried under vacuum.

**Supplementary Figure 103: solid-state synthesis of giant calixarenes - monitoring of the evolution of the solid precursor with time. A): crude reaction media at the end of the 20h refluxing period in octane; B) and C): evolution of the composition of the crude product during heating monitored by 1H NMR spectroscopy; D): MALDI MS analysis of the crude product at the end of the refluxing period. PRUs shown in boxes.**

**Linear oligomers recovery**

The acetone/DMSO filtrate recovered after the low-temperature crystallization of giant calixarenes is evaporated, resulting in a DMSO-only solution. After adding 10% (volume) of ethanol, the solution is kept at 1°C for 3 days, resulting in the formation of a microcrystalline precipitate, recovered by filtration. A combined NMR and MALDI analysis shows this precipitate to be constituted only of linear oligomers.

**Supplementary Figure 104: general formula of the observed linear oligomers**

**Example of linear oligomers recovery from a two-steps giant calixarenes synthesis (CsOH 0.4 equivalent)**

Yield: 15g (14%).

**Supplementary figure 105: ^1^H NMR** (DMSO-d6)

**Supplementary Figure 106: ^13^C NMR** (DMSO-d6)

**Supplementary Figure 107: MALDI MS mass spectra of linear oligomers** (after addition of CF_3_COOK)

The expected m/z value for *p*-(benzyloxy)calix[10]arene is shown in the box.

**Supplementary Figure 108: Size Exclusion Chromatography (SEC) analysis**

**Calixarenes recovery**

The DMSO/EtOH filtrate obtained after the recovery of linear oligomers is precipitated by addition of 1L of methanol. The filtrate is recovered by filtration, washed with methanol, dried under vacuum, and directly analysed. A combined MALDI MS/^1^H NMR analysis shows this filtrate to be constituted only of a mixture of calix[n]arenes, with n=6,7 and 9<n<14.

**Example 1 - calixarenes recovery from a two-steps process (CsOH 0.4 equivalent)**

Yield: 50%.

**Supplementary Figure 109: ^1^H NMR** (DMSO-d6)

**Supplementary Figure 110: MALDI MS mass spectra (after addition of CF_3_COOK). A**

PRUs are shown in the boxes.

**Supplementary Figure 111: SEC analysis.**

**Example 2 - calixarenes recovery from a solid-state synthesis (CsOH 0.4 equivalent / silicone oil as heat transmitter)**

Yield: 25%.

**Supplementary Figure 112: MALDI MS mass spectra (after addition of CF_3_COOK)**

PRUs are shown in boxes.

**SEC analysis**

PRU shown in the boxes.

**Supplementary Figure 113: SEC analysis of recovered calixarenes**

**Supplementary Figure 114: Overall purification process of giant calixarenes (DMSO /acetone based crystallization)**

**Giant calixarenes synthesis from *p*-(tBu)phenol (one-step process)**

**Synthesis**

A 1l two-necked, round bottomed flask fitted with a magnetic stirrer and a dean-stark water collector (fitted with a reflux condenser) is filled with a suspension of 31.5g of *p*-(tBu)phenol (0.21 mol), 15g of paraformaldehyde (0.5 mol) and 30 ml of CsOH (50% w/w aqueous solution, 0.174mol) and 300mL of xylene under argon. The resulting suspension is then refluxed for 18 hours under strong stirring. The resulting deep orange solution is then neutralized with 20ml of 37% aq. HCl and 200 ml of THF under strong stirring. The as obtained light orange solution is then directly analysed by MALDI MS.

**MALDI MS analysis**

The crude material obtained after the neutralization step is directly analysed by MALDI mass spectrometry (cationisation with cesium trifluoroacetate). A complex mixture of products is observed, where calixarenes with a number of phenolic repetition units (PRUs, see boxes) between 9 and 30 are the mains species.

**Supplementary Figure 115: MALDI-MS analysis of the crude product obtained using *p*-(tBu)phenol (PRU shown in boxes).**

**Giant calixarenes synthesis from *p*-(n-heptyl)phenol (two-steps process)**

**Synthesis**

A 250ml three-necked, round bottomed flask fitted with a mechanical stirrer, a dean-stark water collector and a lateral stopper (connected to a bubbler) is filled with a suspension of 25g of n-(heptyl)phenol (0.13 mol), 24 ml of a 37% formaldehyde aqueous solution (0.27 mol) and 20 ml of CsOH (50% w/w aqueous solution, 0.11mol) under argon flushing. The resulting suspension is then brought to reflux for 20min under strong mechanical stirring. The lateral stopper is then opened, allowing for a fast argon stream to flow out of the flask (10 bubbles/second) while still heating, in order to remove water. The reaction media then solidifies as a deep red waxy material. 70mL of xylene are then added, and the resulting solution is refluxed for 15 h. The resulting deep red solution is then neutralized with 11 ml of 37% aq. HCl and 20 ml of THF under strong stirring.

**Analysis of giant calixarenes**

The crude material obtained after the neutralization step is directly analyzed by MALDI mass spectrometry (cationisation with sodium trifluoroacetate). Calixarenes with a number of phenolic repetition units (PRUs, see boxes) between 9 and 30 are the mains species observed.

**Supplementary Figure 116: A) MALDI-MS analysis of the crude product obtained using *p*-(heptyl)phenol; B) zoom (PRUs shown in the boxes).**

**Synthesis of giant calixarenes using chlorobenzene as a solvent (CsOH 0.6 equivalent vs. phenol, one-step)**

Note: the density of chlorobenzene being higher than 1 (1.1), a dean-stark-type apparatus can no longer be used for water removal. The Dean-stark water remover was thus replaced by a drying cartridge filled with oven-dried MgSO_4_.

A 1l, three necked round-bottomed flask was fitted with a dropping funnel loaded with oven-dried MgSO_4_. A reflux condenser was then fixed on top of this funnel. Under argon flushing, the flask was then loaded with 25g of *p*-(benzyloxy)phenol (0,135 mol), 350 ml of chlorobenzene, 8.1 g of paraformaldehyde (0.27 mol), and 15 ml of CsOH (50% water solution, 0.081 mol). The solution was then refluxed under argon and strong mechanical stirring for 13h. After neutralization with 7 mL of aqueous hydrochloric acid in 200 mL of THF, the product was directly analyzed.

**Supplementary Figure 117: crude product analysis - ^1^H NMR** (DMSO-d6).

Note: Peaks market with green asterisks are belonging to *p*-(benzyloxy)bis(homoxa)calix[4]arene ^[6](#_ENREF_6)^.

**Supplementary f118**

The MALDI MS (Matrix-Assisted Laser-induced Desorption-Ionisation Mass Spectrometry) analysis of the crude product is shown on Supplementary Figure 118. Only calixarenes are observed (number of repeating units shown in the boxes), associated with satellite signals. Supplementary Figure 119 also displays a zoom in order to make clearer the analysis of these satellite signals. These extra signals are produced during the MS analysis, due to the formation of one, or two Na^+^ adducts (M+23 and M+22 peaks), and K^+^ ones (M+39), solid red arrows. Moreover, a second series of signals is observed, corresponding to laser-induced debenzylation reactions (M-91, green arrows).

**See zoom on the following page**

**Supplementary Figure 119: MALDI-MS analysis of the product obtained using chlorobenzene as the solvent. Green arrows: photoinduced debenzylations; red arrows: H^+^ 🡪 Na^+^ and K^+^ exchanges.**

**Supplementary figure 120: crude product - SEC analysis**

**Supplementary figure 121: SEC-MALS analysis**

**Supplementary Figure 122: Chlorobenzene / xylene comparison (crude products).**

Note: for both syntheses: i) CsOH, 0.6 equivalent vs. phenol, ii) one-step process.

**Supplementary Figure 123: Comparison between crude product from three different staring phenols (CsOH 0.8 equivalent vs. phenol, one-step in xylene each)**

***p*-(octyloxy)phenol synthesis**

**Synthesis:**

A 500ml, three-necked round bottomed flask fitted with a dean-stark water collector, a refrigerant and a magnetic stirrer is loaded with 25g of *p*-(octyloxy)phenol (0.113 mol.), 7.5 g of solid paraformaldehyde (0.226 mol.), 5.5 ml of a 50% weight aqueous RbOH solution (0.045 mol.) and 270 ml of xylene is heated at reflux for 8 hours.

After addition of 200ml of THF, the resulting suspension is neutralized with HCl under vigorous stirring, and evaporated to dryness.

**Analysis:**

**Supplementary Figure 124: crude product - ^1^H NMR analysis** (DMSO-d6).

**Supplementary Figure 125: crude product - MALDI-MS analysis (Na^+^/K^+^ cationised)**

**Supplementary Figure 126: crude product - Size Exclusion Chromatography (SEC) analysis**

Number of constituting phenolic cycles indicated on the chromatogram for each peak.

**Supplementary Figure 127: MALDI-MS analysis of a giant calixarenes synthesis run with 0.8 equivalent of KOH vs. phenol (two-steps process).**

This acetamide-derivatized sample shows large amounts of linear oligomers (red asterisks), along with calixarenic species (PRU shown in the boxes).

**Supplementary References**

1 Gutsche, C. D. & Stewart, D. R. *J. Am. Chem. Soc.* **121**, 4136 (1999).

2 Hanton, S. D. *Chem. Rev.* **101**, 527 (2000).

3 Striegel, A. M., Yau, W. W., Kirkland, J. J. & Bly, D. D. *Modern Size Exclusion Chromatography. Second ed.*, 496 (John Wiley & Sons, 2009).

4 Wyatt, P. J. Light scattering and the absolute characterization of macromolecules. . *Analytica Chimica Acta* **272**, 1 (1993).

5 De Gennes, P.-G. *Scaling concept in polymer physics*. (Cornell University Press: Ithaca, NY, 1979).

6 Huc, V. *et al.* *Eur. J. Org. Chem.* **11**, 6186 (2010).

1. [↑](#footnote-ref-4)
